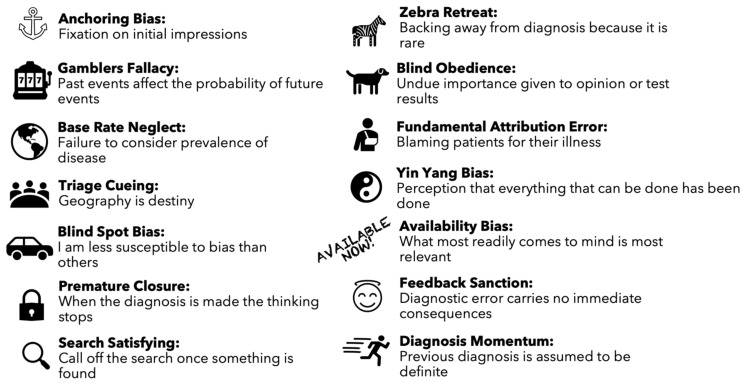# Cognitive Errors and Debiasing

**DOI:** 10.21980/J84W96

**Published:** 2025-07-31

**Authors:** Joshua Ginsburg

**Affiliations:** * University of Texas Southwestern, Department of Emergency Medicine, Dallas, Tx

## Abstract

**Audience:**

Although this lecture was given to first-year residents, it is also appropriate for upper-level residents, medical students, fellows, and faculty.

**Introduction:**

Medical errors are largely due to errors of cognition rather than lack of knowledge.^1^ The cognitive processes that underlie these errors are often explained using Dual Process Theory, which posits that we engage in either fast, intuitive, low-effort System 1 thinking or slow, analytical, high-effort System 2 thinking. Although System 1 thinking is crucial for efficient emergency medicine practice, it is susceptible to the biases that cause cognitive errors. Research to date is mixed regarding the effect of educational interventions aimed at reducing cognitive bias but tends to show a benefit to cognitive bias training over a variety of outcome measures.^2^ Many experts therefore believe that physicians should be taught about cognitive biases and debiasing strategies in an effort to reduce medical errors.^3^,^4^

**Educational Objectives:**

By the end of this lecture, learners should be able to, 1) Define dual process theory, 2) identify common cognitive biases, 3) recognize high-risk situations for cognitive errors, and 3) discuss debiasing strategies and integrate one strategy into your workflow.

**Educational Methods:**

This interactive lecture was created in PowerPoint and delivered in-person to 14 first-year residents during their “Intern Curriculum,” a monthly meeting separate from the residency-wide conference. The lecture took 30 minutes to deliver.

**Research Methods:**

Residents responded to pre- and post-lecture Likert scale surveys regarding their knowledge of cognitive biases and debiasing strategies, as well as a post-lecture survey regarding the quality of the lecture, the relevance of the content, and the likelihood of making changes to their practice based on the lecture.

**Results:**

A total of 14 residents responded to the survey, and all residents completed both the pre-lecture and post-lecture questions. In the pre-lecture survey, 35.7% (5) of participants reported that they had good or extensive knowledge of cognitive biases, and 7.1% (1) of participants reported that they had good or extensive knowledge of debiasing strategies. In the post-lecture survey, 85.7% (12) of participants reported that they had good or extensive knowledge of cognitive biases, and 78.6% (11) of participants reported that they had good or extensive knowledge of debiasing strategies. All (14) participants felt the lecture was of good or excellent quality, 92.9% (13) felt it was very or extremely relevant to them as emergency medicine physicians, and 100% (14) reported they were likely to make changes to their practice based on this lecture.

**Discussion:**

The results of the survey show that residents perceived increased knowledge of both cognitive errors and debiasing strategies after attending this lecture. The lecture was rated highly, was found to be relevant to practice, and was likely to change practice going forward for most learners. These results suggest that an interactive lecture may have an important role in introducing residents to the concepts of cognitive errors and debiasing.

**Topics:**

Cognitive bias, bias, debiasing, errors.

## USER GUIDE

**Table:** 

List of Resources:	
Abstract	1
User Guide	3
Cognitive Errors and Debiasing Lecture	5
Cognitive Errors and Debiasing Handout	6

**Learner Audience:**

Medical Students, Interns, Junior Residents, Senior Residents, Fellows, Faculty

**Time Required for Implementation:** 30 minutes

**Recommended number of learners per instructor:**

In order to allow for audience interaction with each other and with the presenter, this lecture works best with 6–16 learners.

**Topics:**

Cognitive bias, bias, debiasing, errors.

**Objectives:**

By the end of this lecture, learners should be able to:

1. Define dual process theory.
2. Identify common cognitive biases.
3. Recognize high-risk situations for cognitive errors.
4. Discuss debiasing strategies and integrate one strategy into your workflow.

### Linked objectives and methods

Objective 1 is achieved by participating in the matching activity, in which a case is presented and learners identify the cognitive biases at play. Objective 2 is achieved through the think-pair-share activity, in which learners and their neighbors come up with high-risk situations for cognitive errors and share them with the group. Objective 3 is achieved through the final discussion regarding debiasing strategies that the learners currently use or plan to try. The lecture format was chosen for ease of implementation into the existing didactic curriculum but avoids common pitfalls of traditional lectures by incorporating case-based and interactive elements, which capitalize on problem-based learning, active learning theory, and collaborative learning theory.

### Recommended pre-reading for instructor

- Croskerry P, Singhal G, Mamede S. Cognitive debiasing 1: origins of bias and theory of debiasing. *BMJ Qual Saf*. 2013;22 Suppl 2(Suppl 2):ii58–ii64.
  doi:10.1136/bmjqs-2012-001712
- Croskerry P, Singhal G, Mamede S. Cognitive debiasing 2: impediments to and strategies for change. *BMJ Qual Saf*. 2013;22 Suppl 2(Suppl 2):ii65–ii72.
  doi:10.1136/bmjqs-2012-001713
- Morganstern J. Cognitive errors in medicine: The common errors. First10EM.com.
https://first10em.com/cognitive-errors/

### Learner responsible content (LRC, optional)

- Prior to this lecture, learners should review the single page summary of some of the more common cognitive biases (attached).

### Results and tips for successful implementation

This lecture is best delivered in an in-person lecture environment that is conducive to pairing of learners and subsequent discussion. In our implementation, residents were arranged in a semicircle around the presenter, with the PowerPoint projected behind the presenter. This allowed for residents to easily view the lecture material, pair up in groups of two, and share the results of their discussions with the entire group.

Prior to the lecture, residents answered two Likert scale survey questions regarding their knowledge of cognitive biases and debiasing strategies. Immediately after the lecture, residents answered those same two questions in addition to three questions regarding the quality of the lecture, the relevance of the content, and the likelihood of making changes to their practice based on the lecture. All 14 residents completed both the pre-lecture and post-lecture questions. In the pre-lecture survey, 35.7% (5) of participants reported that they had good or extensive knowledge of cognitive biases, and 7.1% (1) of participants reported that they had good or extensive knowledge of debiasing strategies. In the post-lecture survey, 85.7% (12) of participants reported that they had good or extensive knowledge of cognitive biases, and 78.6% (11) of participants reported that they had good or extensive knowledge of debiasing strategies. All (14) participants felt the lecture was of good or excellent quality, 92.9% (13) felt it was very or extremely relevant to them as emergency medicine physicians, and 100% (14) reported they were likely to make changes to their practice based on this lecture.

### Assessment (optional)

This lecture includes a matching component, in which learners are asked to identify out loud the cognitive bias at play in 14 brief cases of cognitive errors.

### Technology necessary

Lecturers will need a computer that can run PowerPoint and a projector to display the content.

## Supplementary Information



## Figures and Tables

**Figure:**
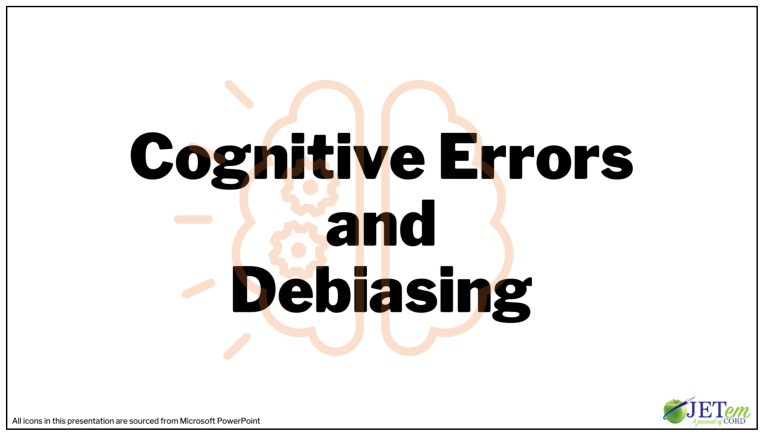
Please see associated PowerPoint file

**Figure:**