# Supplementary material for: Cognitive Errors and Debiasing
Source: J Educ Teach Emerg Med. 2025 Jul 31;10(3):L1–6. doi: 10.21980/J84W96 (PMC12320998; doi:10.21980/J84W96)
Supplement: Supplementary file 1 [file 10-3-L1-Supp1.pptx]

## Slide 1
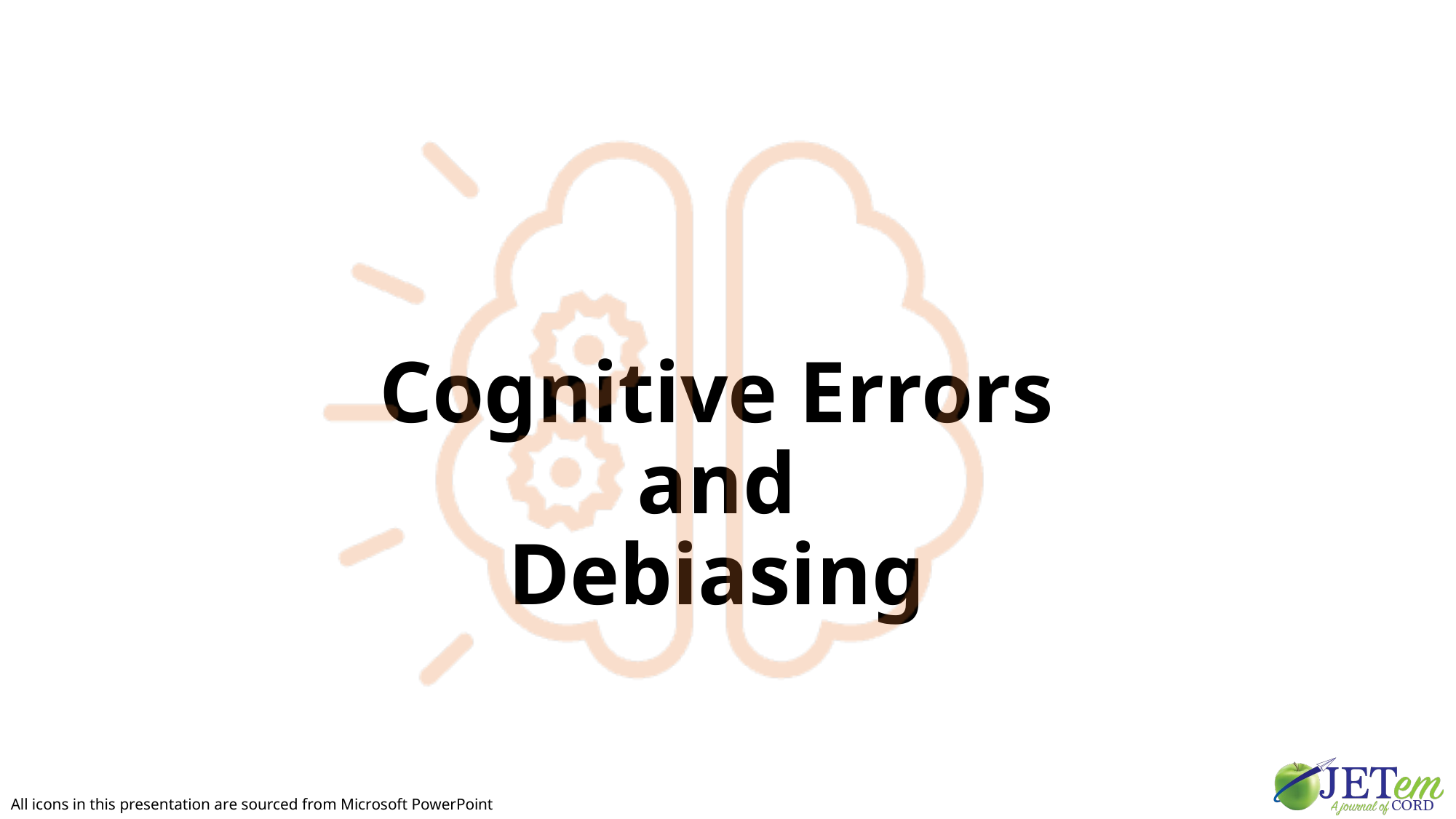

# Cognitive Errors and Debiasing
All icons in this presentation are sourced from Microsoft PowerPoint

## Slide 2
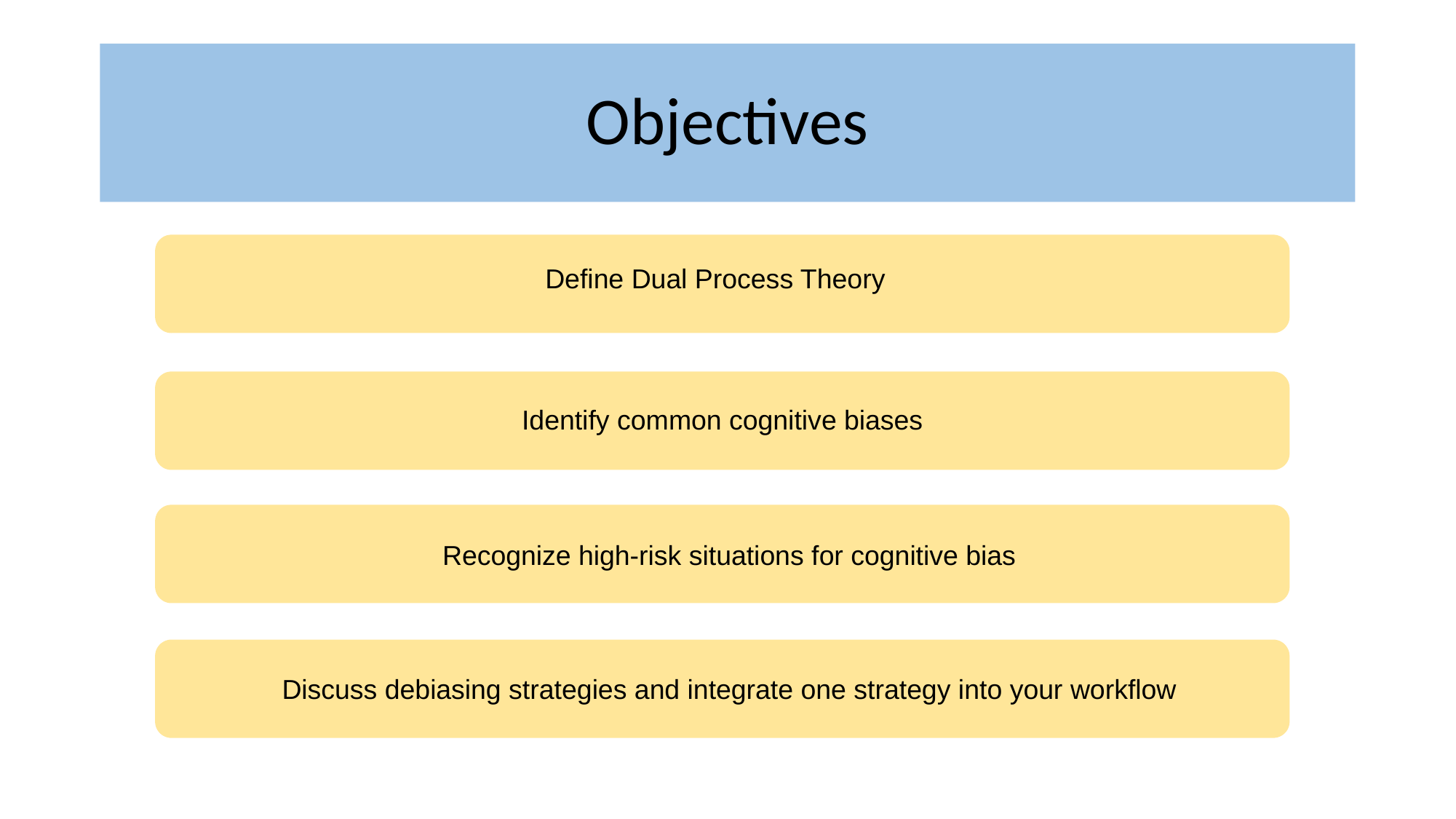

# Objectives
Define Dual Process Theory
Identify common cognitive biases
Recognize high-risk situations for cognitive bias
Discuss debiasing strategies and integrate one strategy into your workflow

## Slide 3
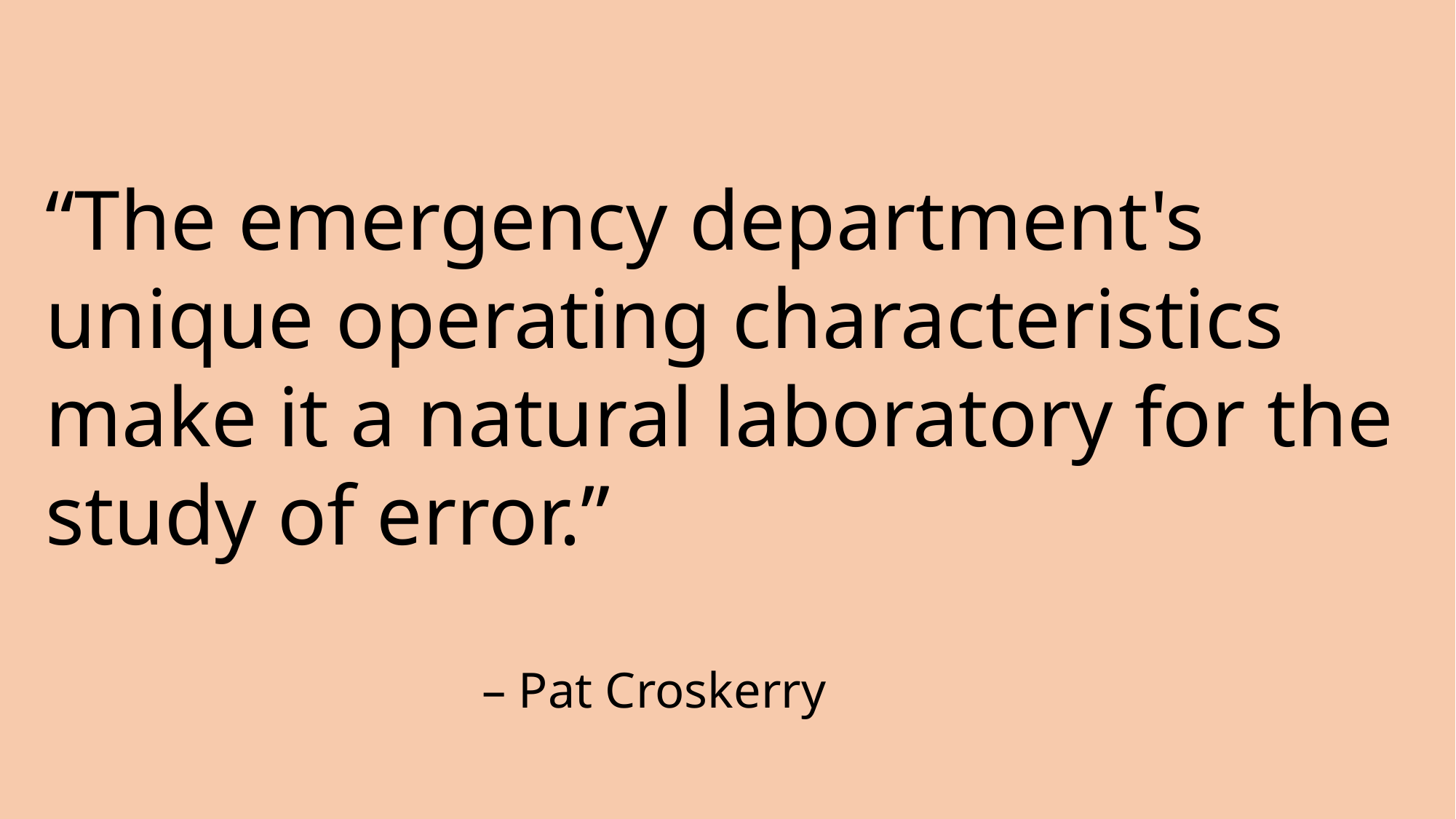

“The emergency department's unique operating characteristics make it a natural laboratory for the study of error.”
																– Pat Croskerry

## Slide 4
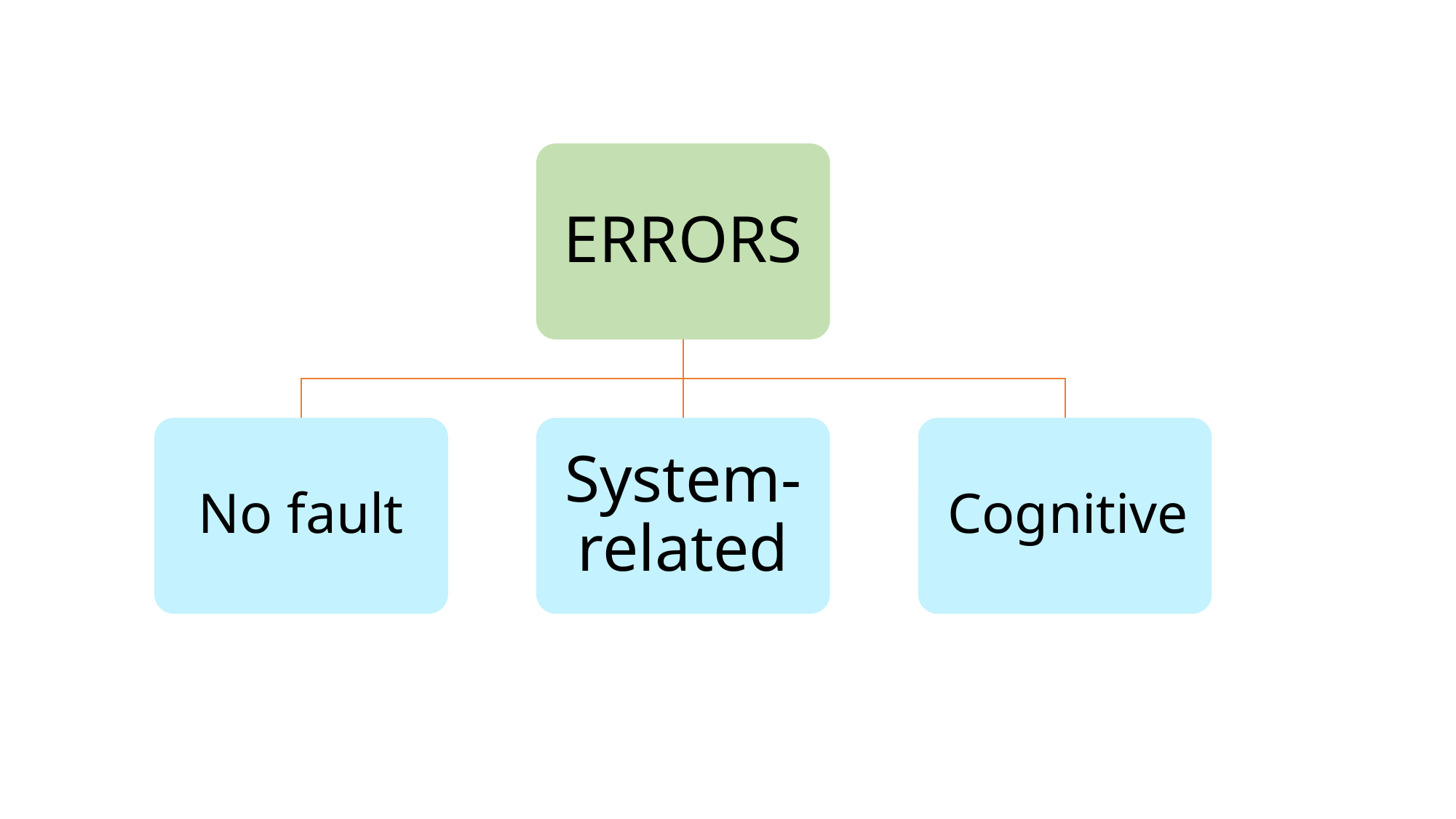

ERRORS
No fault
System-related
Cognitive

## Slide 5
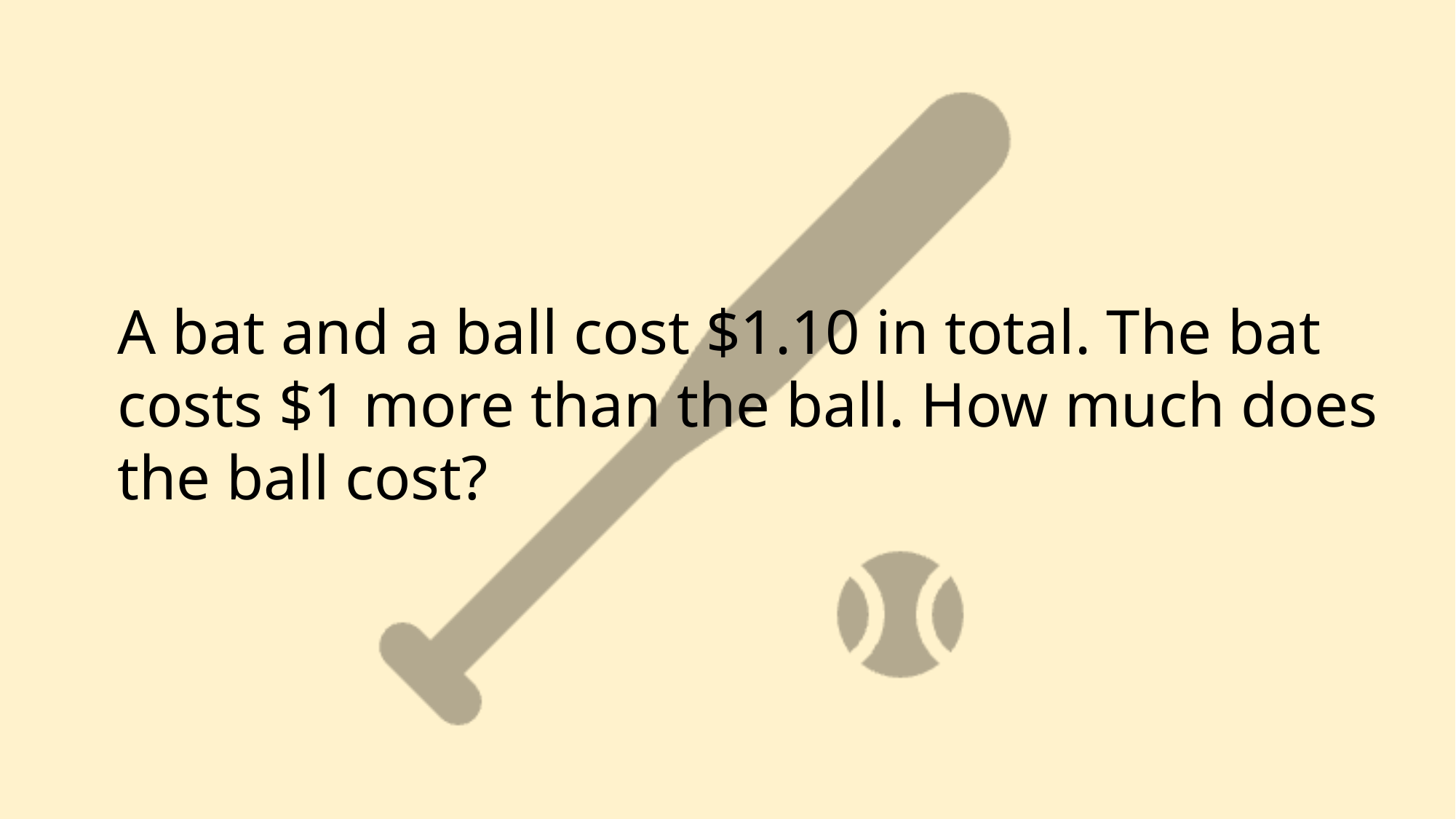

A bat and a ball cost $1.10 in total. The bat costs $1 more than the ball. How much does the ball cost?

## Slide 6
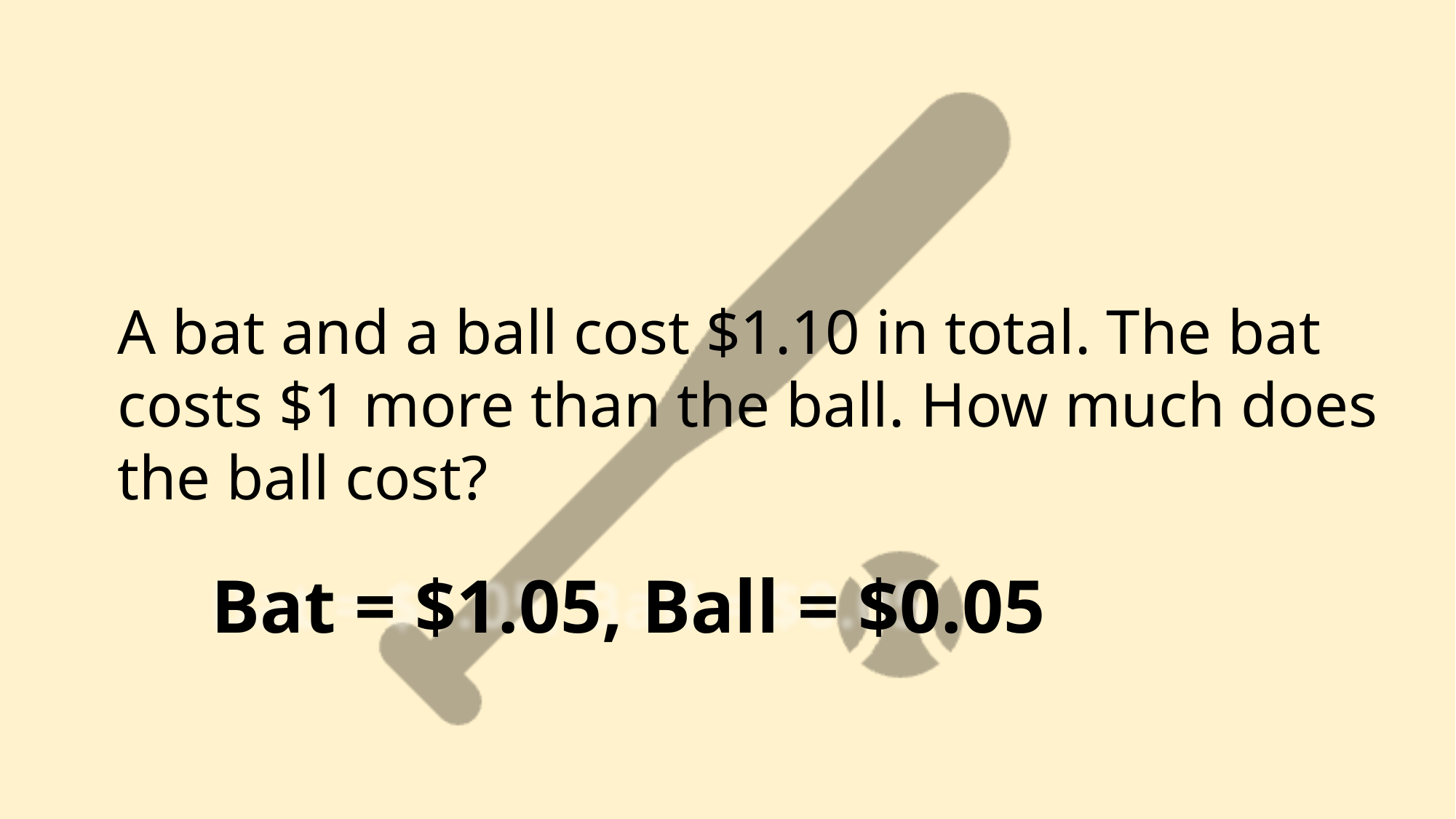

A bat and a ball cost $1.10 in total. The bat costs $1 more than the ball. How much does the ball cost?
Bat = $1.05, Ball = $0.05

## Slide 7
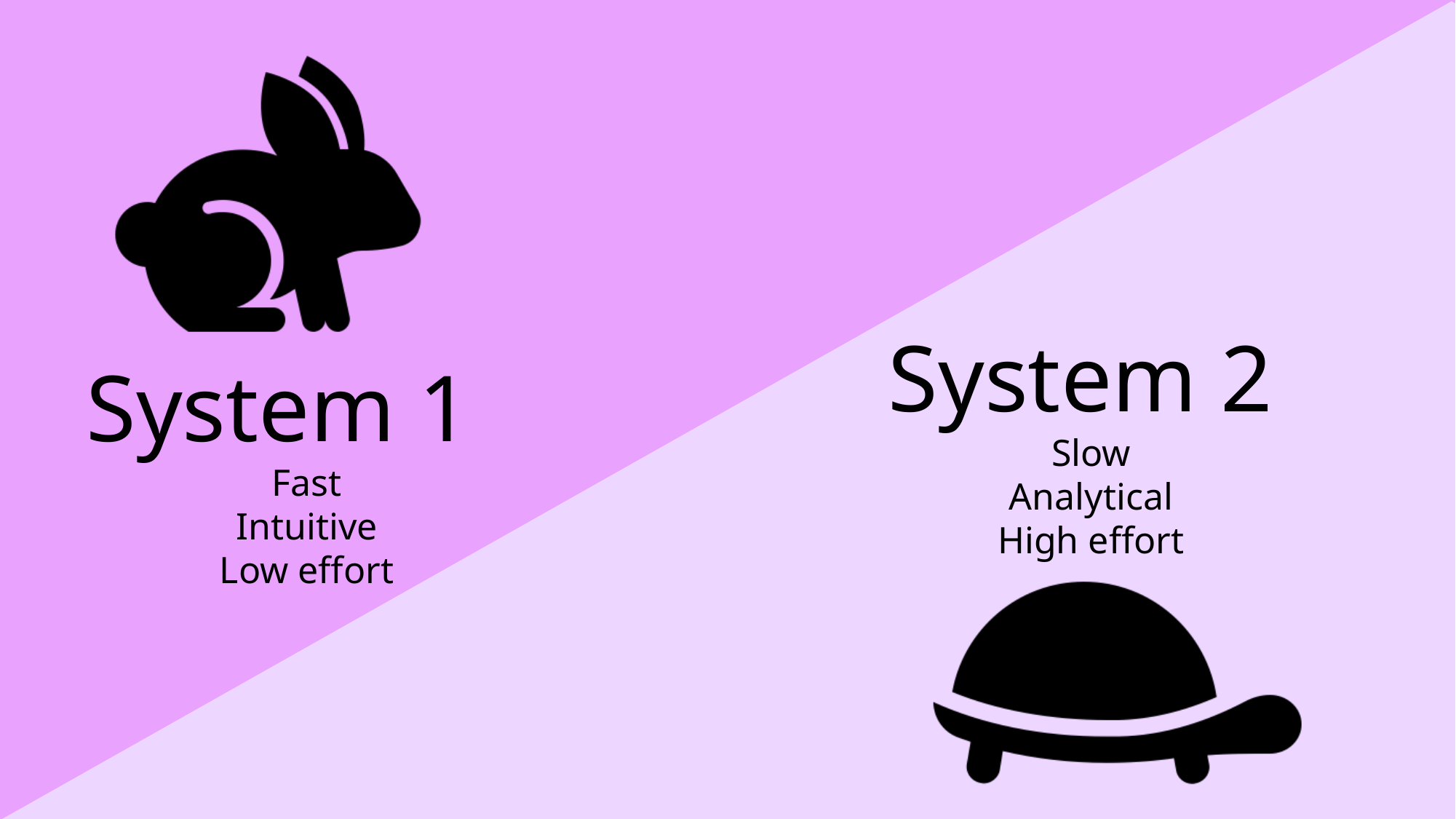

System 2
Slow
Analytical
High effort
System 1
Fast
Intuitive
Low effort

## Slide 8
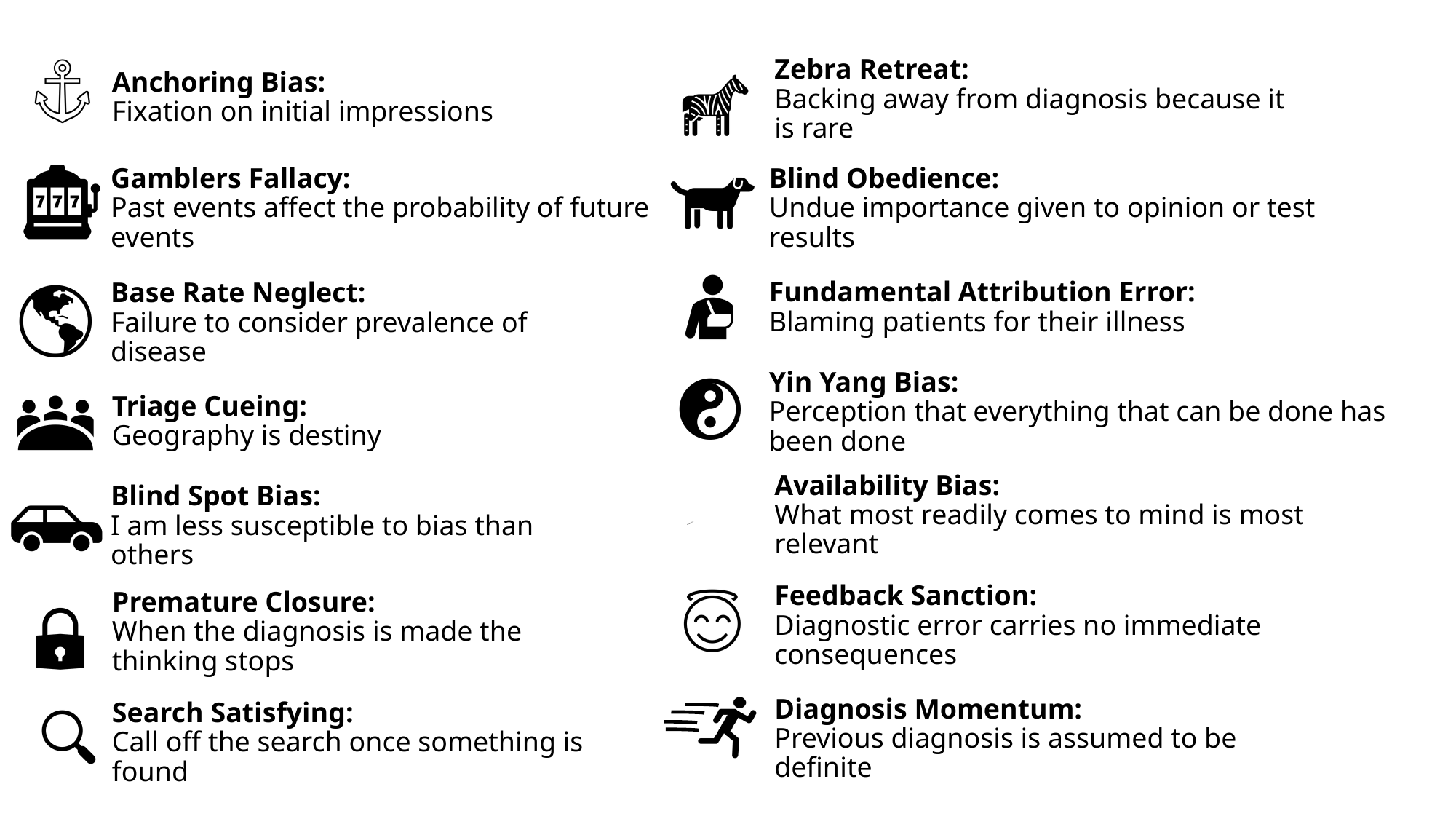

# Anchoring Bias: Fixation on initial impressions
Zebra Retreat:
Backing away from diagnosis because it is rare
Gamblers Fallacy:
Past events affect the probability of future events
Blind Obedience:
Undue importance given to opinion or test results
Fundamental Attribution Error:
Blaming patients for their illness
Base Rate Neglect:
Failure to consider prevalence of disease
Yin Yang Bias:
Perception that everything that can be done has been done
Triage Cueing:
Geography is destiny
Availability Bias:
What most readily comes to mind is most relevant
Blind Spot Bias:
I am less susceptible to bias than others
AVAILABLE NOW!
Feedback Sanction:
Diagnostic error carries no immediate consequences
Premature Closure:
When the diagnosis is made the thinking stops
Diagnosis Momentum:
Previous diagnosis is assumed to be definite
Search Satisfying:
Call off the search once something is found

## Slide 9
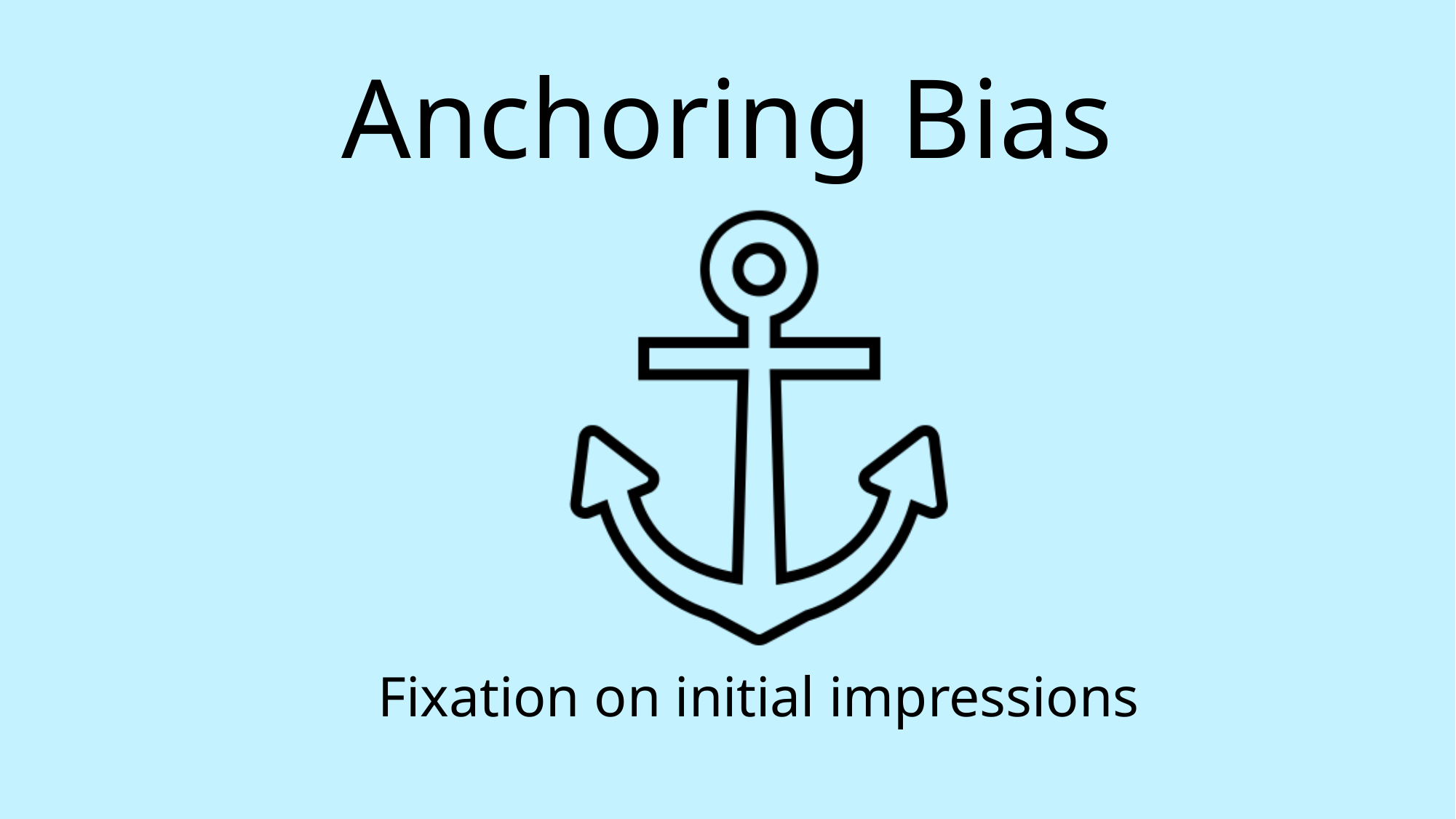

# Anchoring Bias
Fixation on initial impressions

## Slide 10
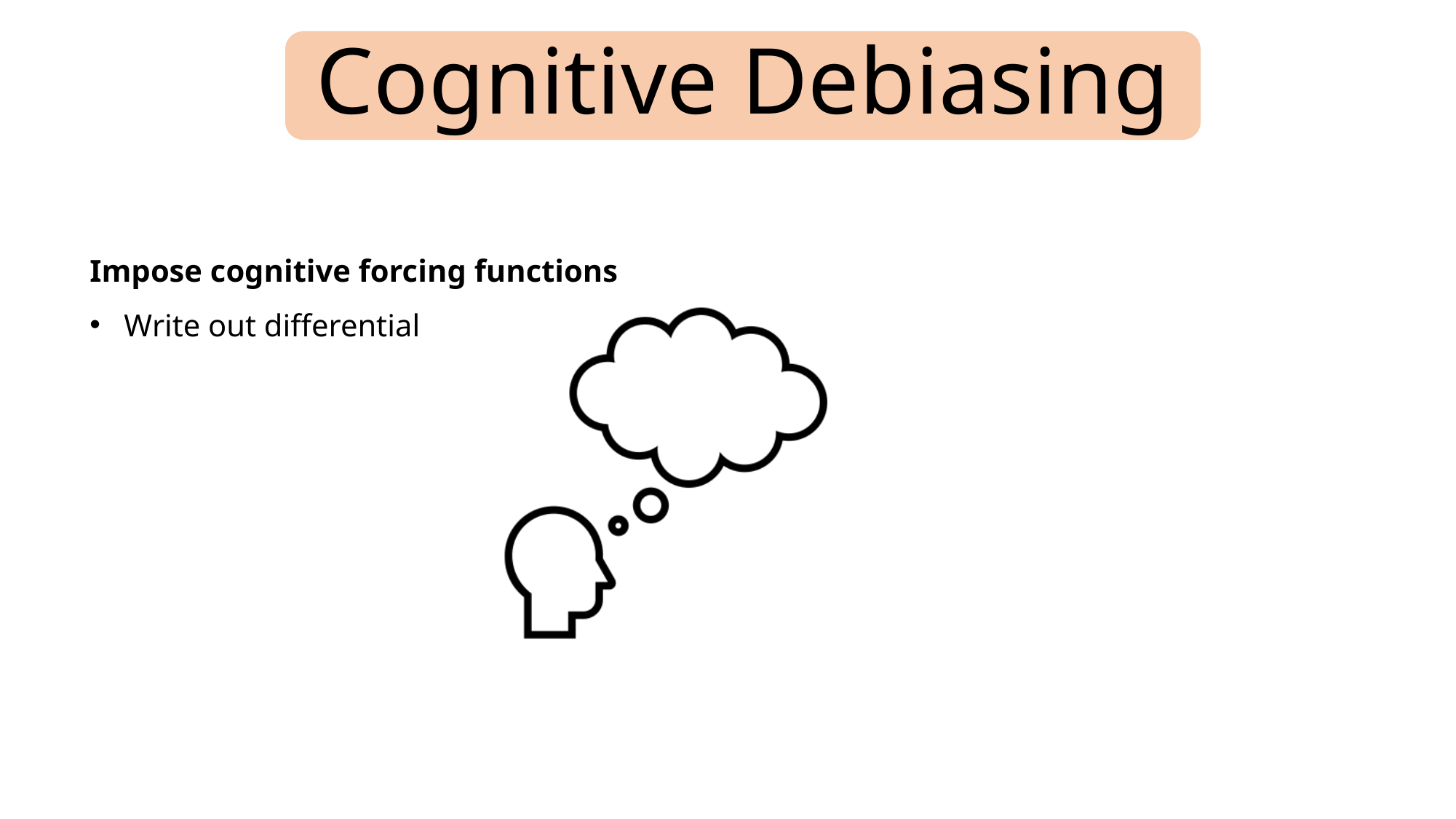

# Cognitive Debiasing
Impose cognitive forcing functions
Write out differential

## Slide 11
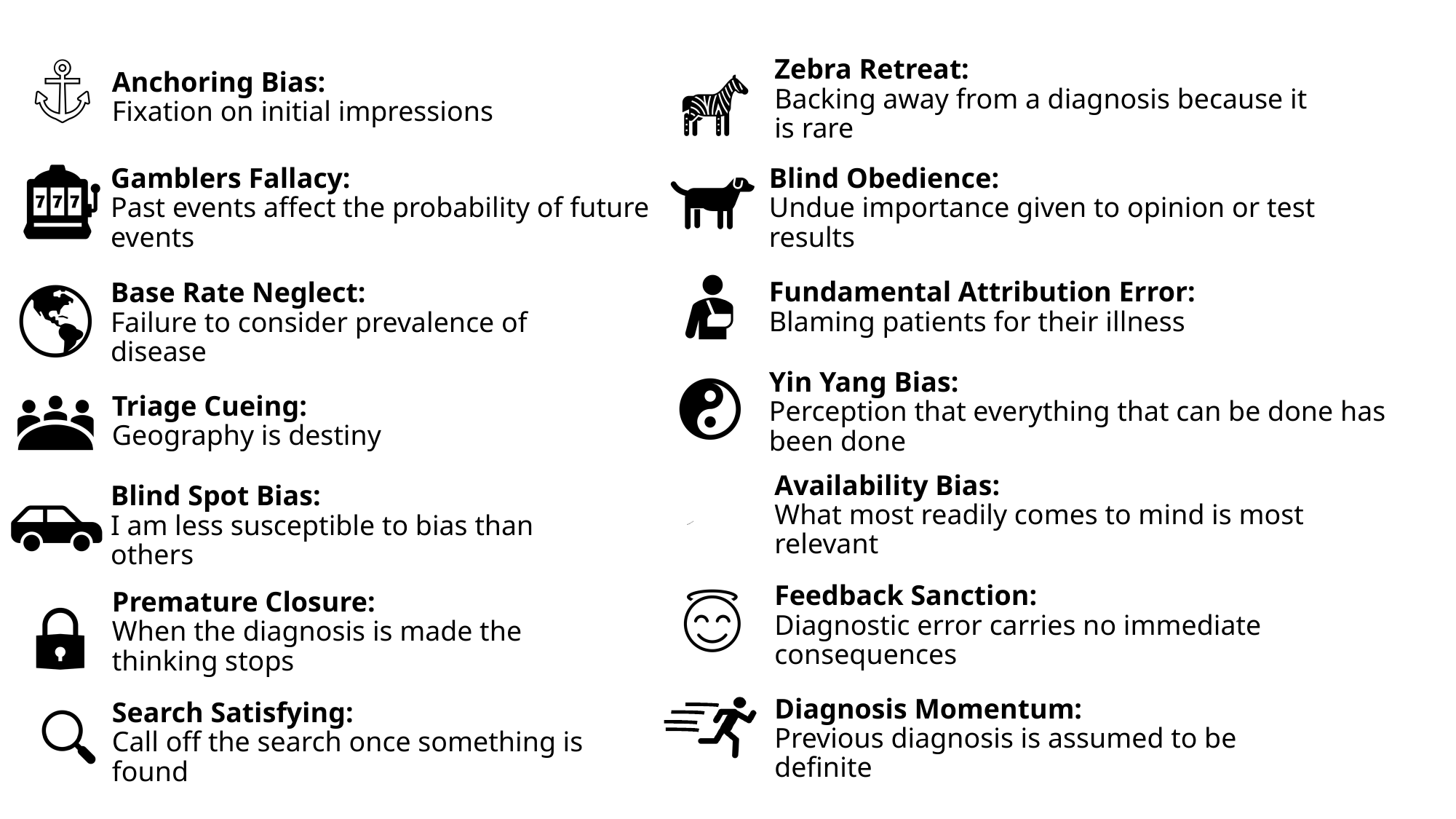

# Anchoring Bias: Fixation on initial impressions
Zebra Retreat:
Backing away from a diagnosis because it is rare
Gamblers Fallacy:
Past events affect the probability of future events
Blind Obedience:
Undue importance given to opinion or test results
Fundamental Attribution Error:
Blaming patients for their illness
Base Rate Neglect:
Failure to consider prevalence of disease
Yin Yang Bias:
Perception that everything that can be done has been done
Triage Cueing:
Geography is destiny
Availability Bias:
What most readily comes to mind is most relevant
Blind Spot Bias:
I am less susceptible to bias than others
AVAILABLE NOW!
Feedback Sanction:
Diagnostic error carries no immediate consequences
Premature Closure:
When the diagnosis is made the thinking stops
Diagnosis Momentum:
Previous diagnosis is assumed to be definite
Search Satisfying:
Call off the search once something is found

## Slide 12
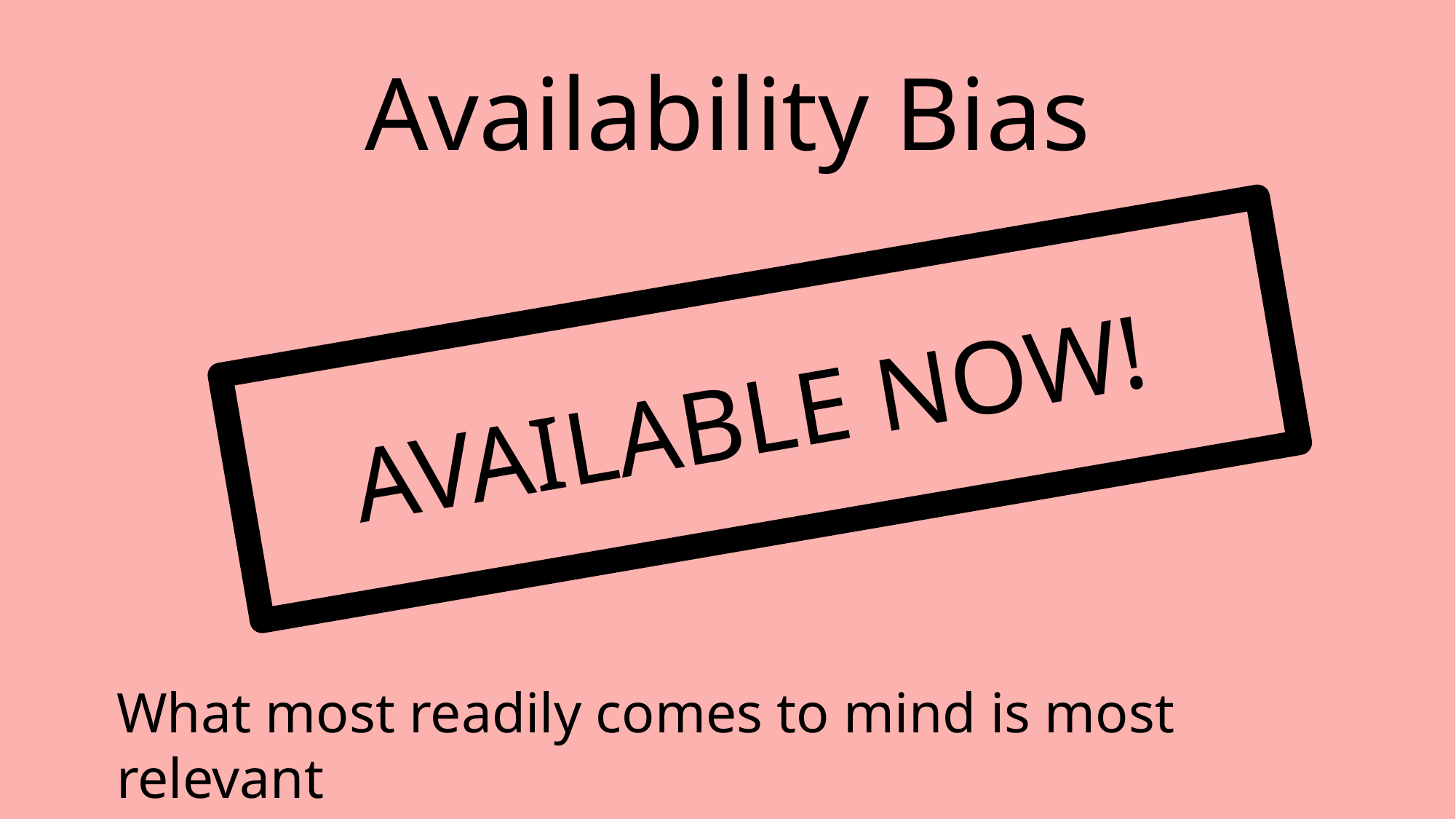

# Availability Bias
AVAILABLE NOW!
What most readily comes to mind is most relevant

## Slide 13
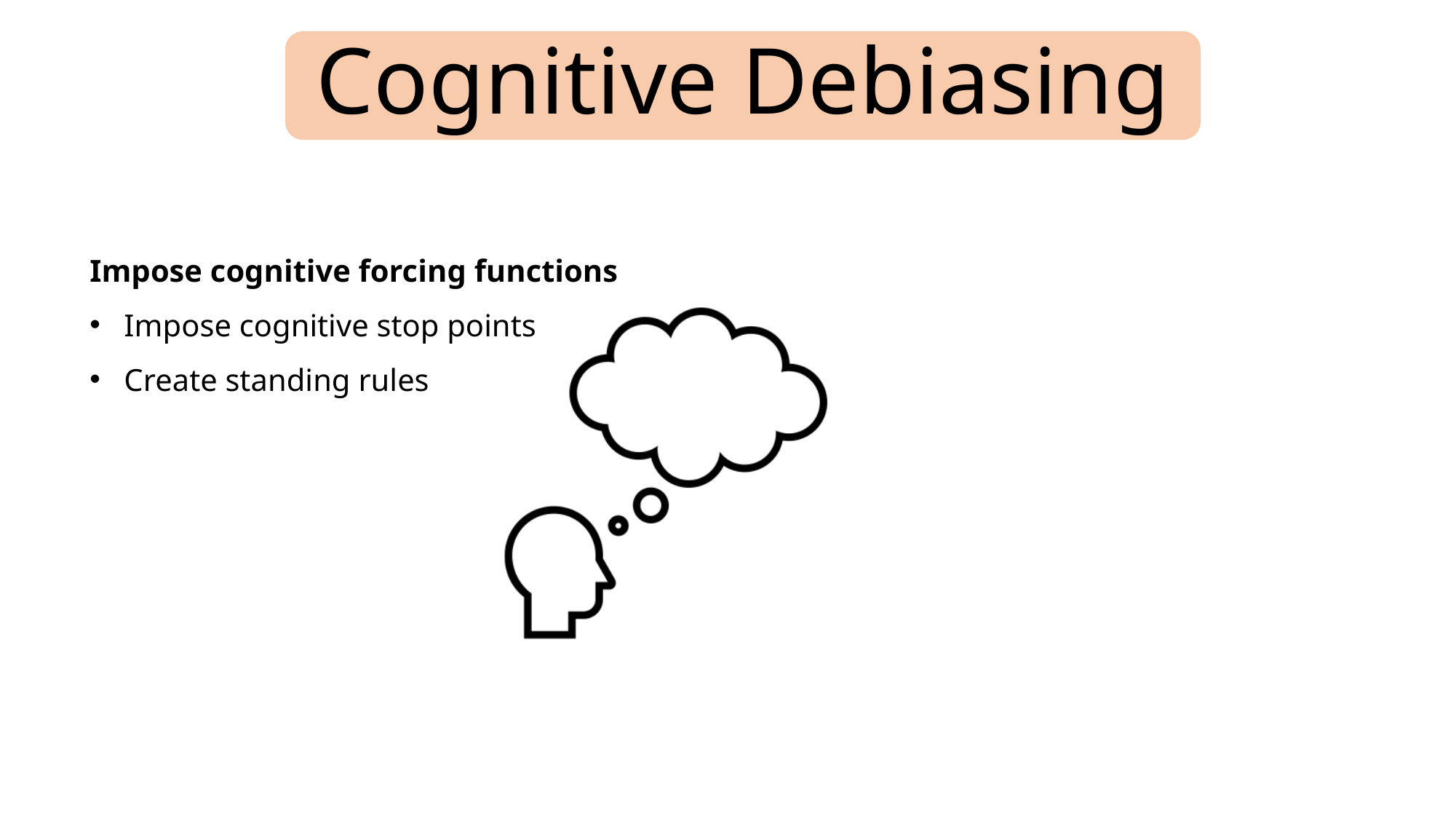

# Cognitive Debiasing
Impose cognitive forcing functions
Impose cognitive stop points
Create standing rules

## Slide 14
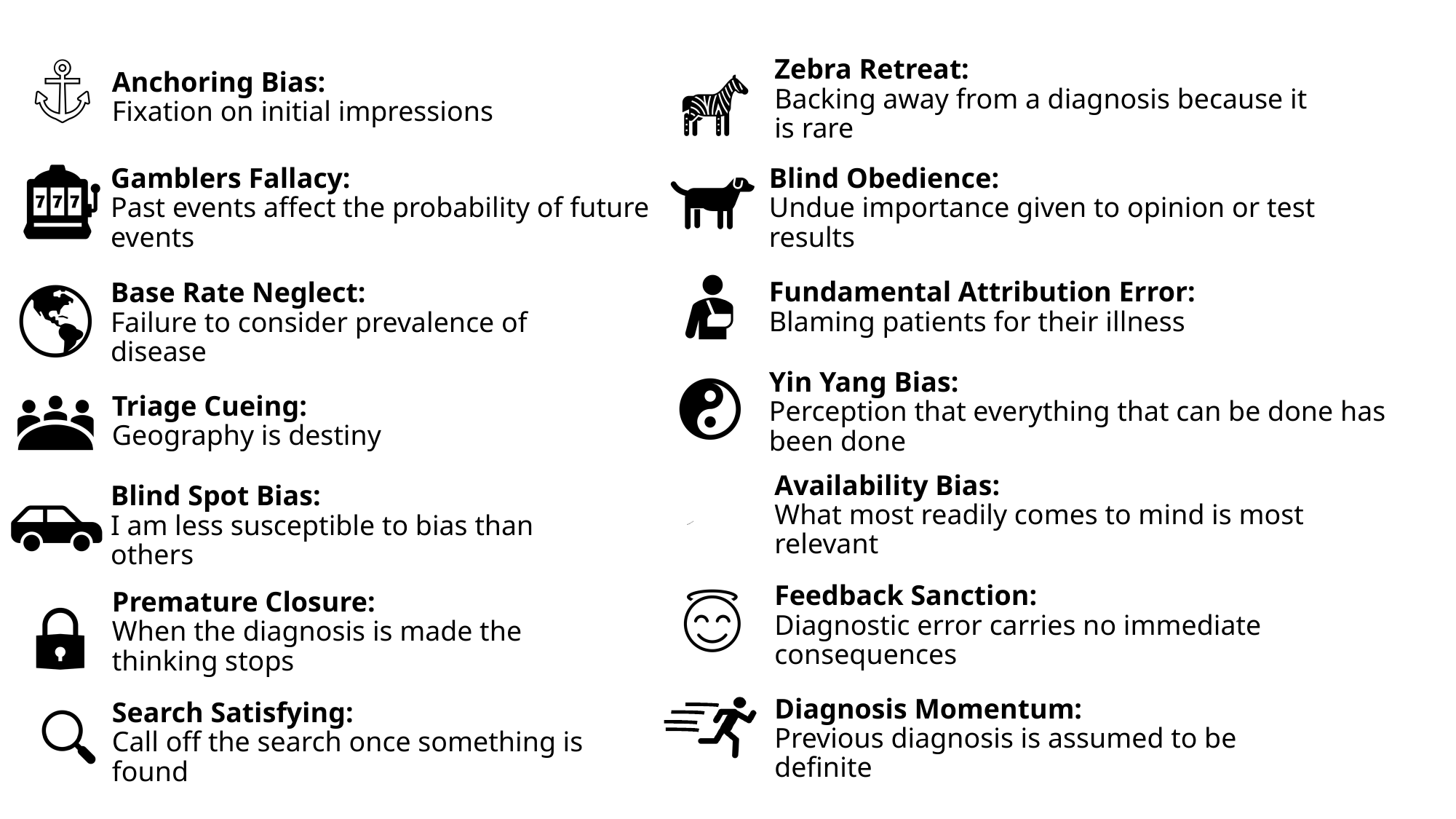

# Anchoring Bias: Fixation on initial impressions
Zebra Retreat:
Backing away from a diagnosis because it is rare
Gamblers Fallacy:
Past events affect the probability of future events
Blind Obedience:
Undue importance given to opinion or test results
Fundamental Attribution Error:
Blaming patients for their illness
Base Rate Neglect:
Failure to consider prevalence of disease
Yin Yang Bias:
Perception that everything that can be done has been done
Triage Cueing:
Geography is destiny
Availability Bias:
What most readily comes to mind is most relevant
Blind Spot Bias:
I am less susceptible to bias than others
AVAILABLE NOW!
Feedback Sanction:
Diagnostic error carries no immediate consequences
Premature Closure:
When the diagnosis is made the thinking stops
Diagnosis Momentum:
Previous diagnosis is assumed to be definite
Search Satisfying:
Call off the search once something is found

## Slide 15
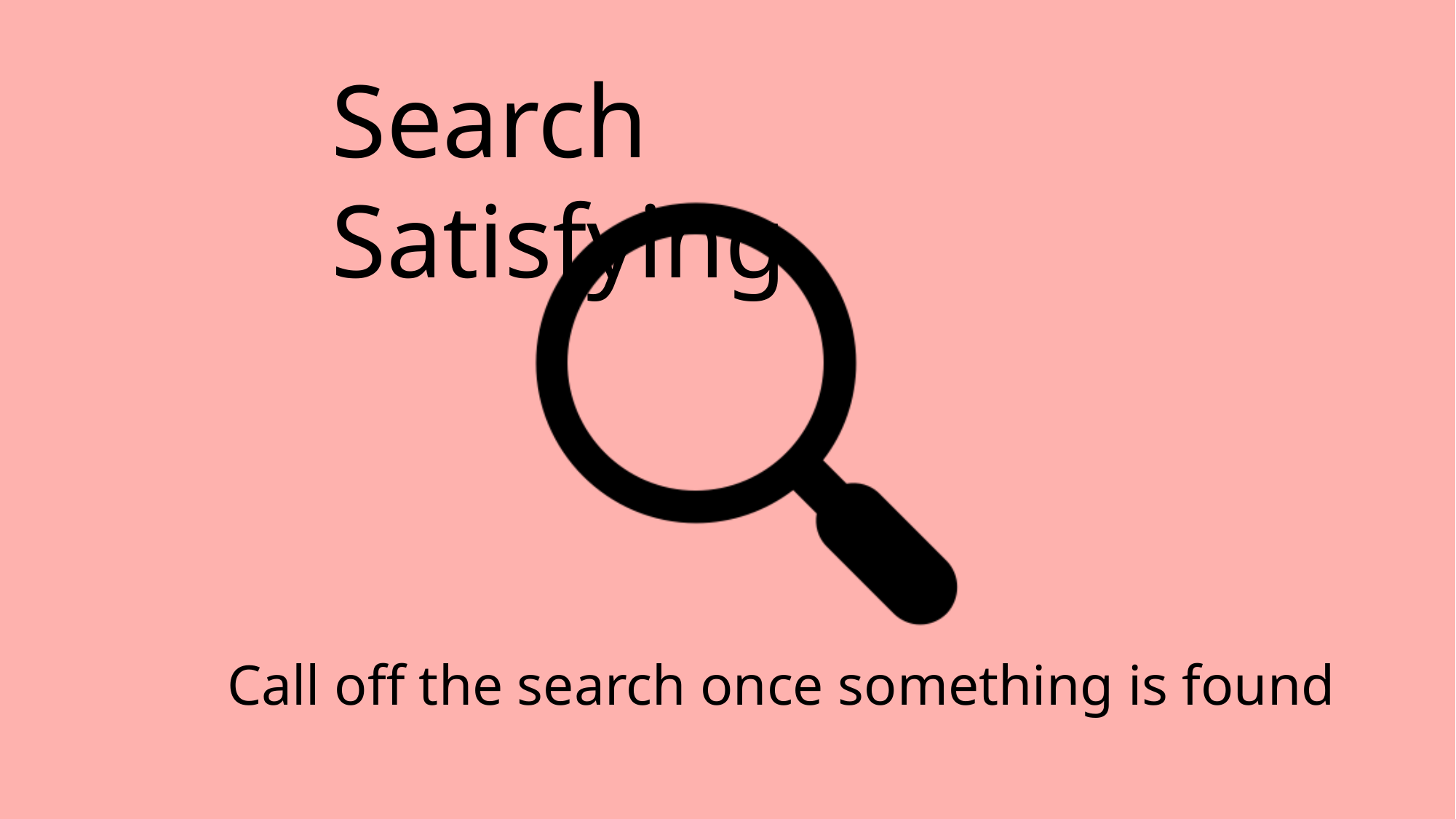

Search Satisfying
Call off the search once something is found

## Slide 16
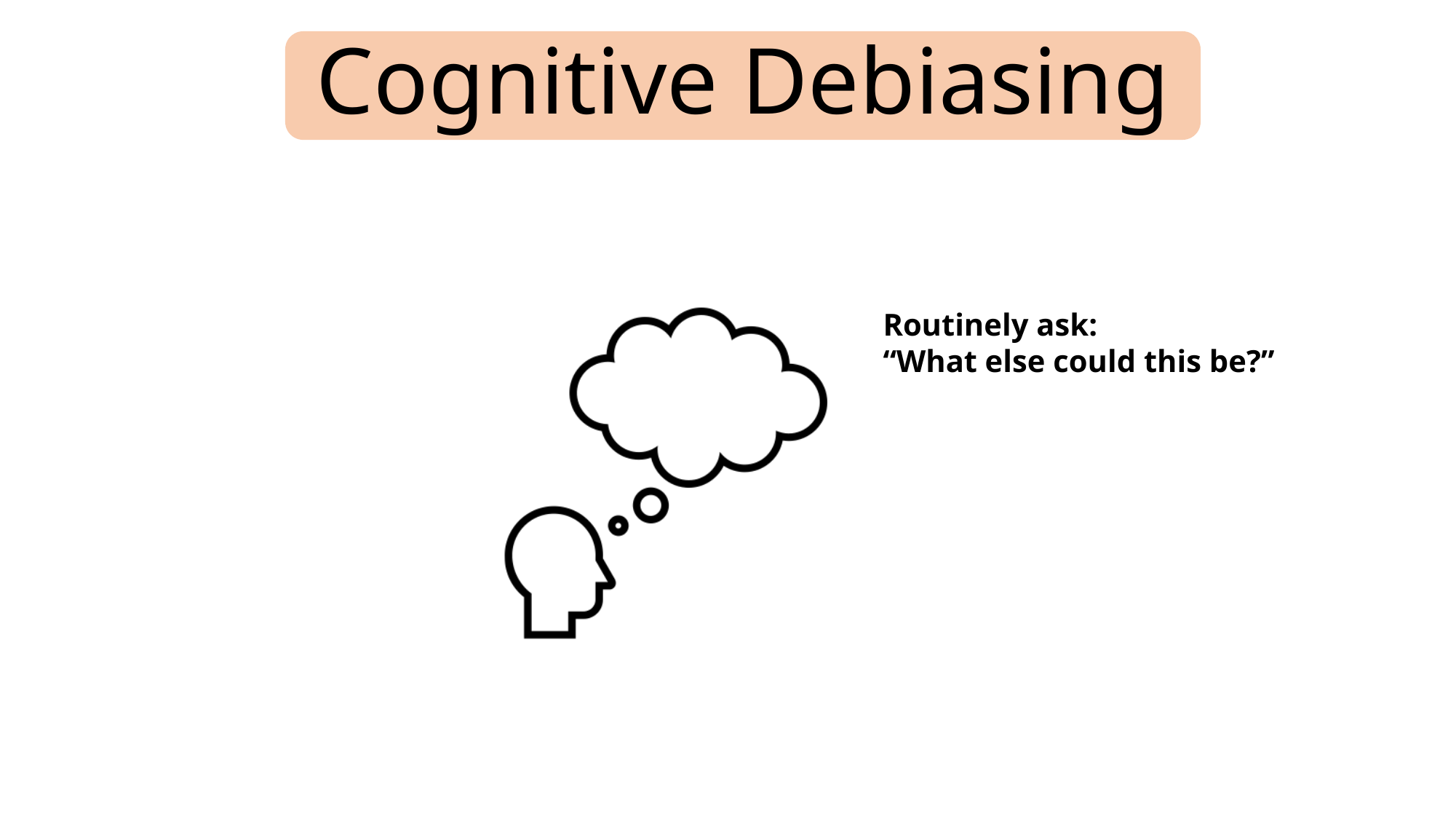

# Cognitive Debiasing
Routinely ask:
“What else could this be?”

## Slide 17
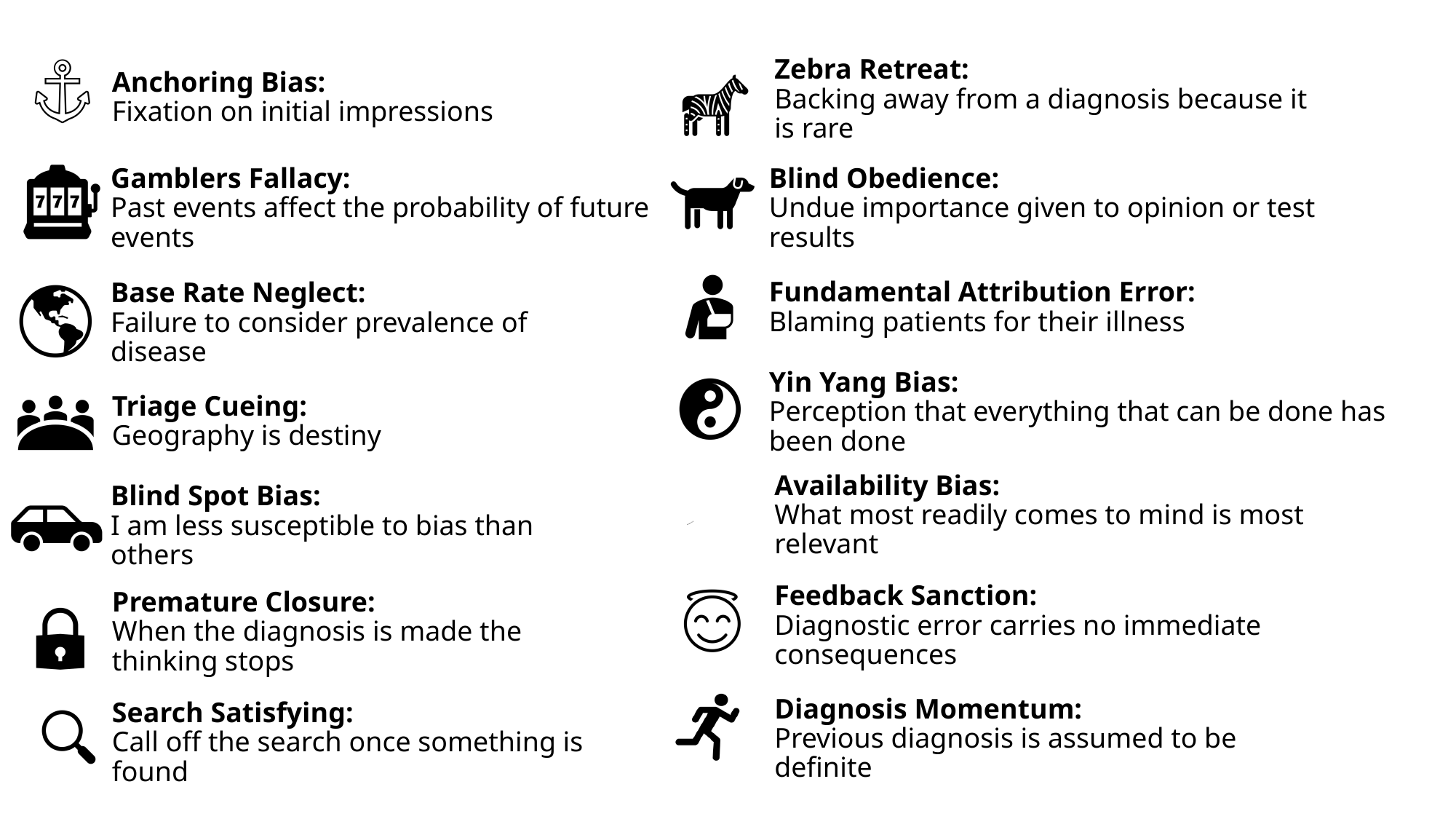

# Anchoring Bias: Fixation on initial impressions
Zebra Retreat:
Backing away from a diagnosis because it is rare
Gamblers Fallacy:
Past events affect the probability of future events
Blind Obedience:
Undue importance given to opinion or test results
Fundamental Attribution Error:
Blaming patients for their illness
Base Rate Neglect:
Failure to consider prevalence of disease
Yin Yang Bias:
Perception that everything that can be done has been done
Triage Cueing:
Geography is destiny
Availability Bias:
What most readily comes to mind is most relevant
Blind Spot Bias:
I am less susceptible to bias than others
AVAILABLE NOW!
Feedback Sanction:
Diagnostic error carries no immediate consequences
Premature Closure:
When the diagnosis is made the thinking stops
Diagnosis Momentum:
Previous diagnosis is assumed to be definite
Search Satisfying:
Call off the search once something is found

## Slide 18
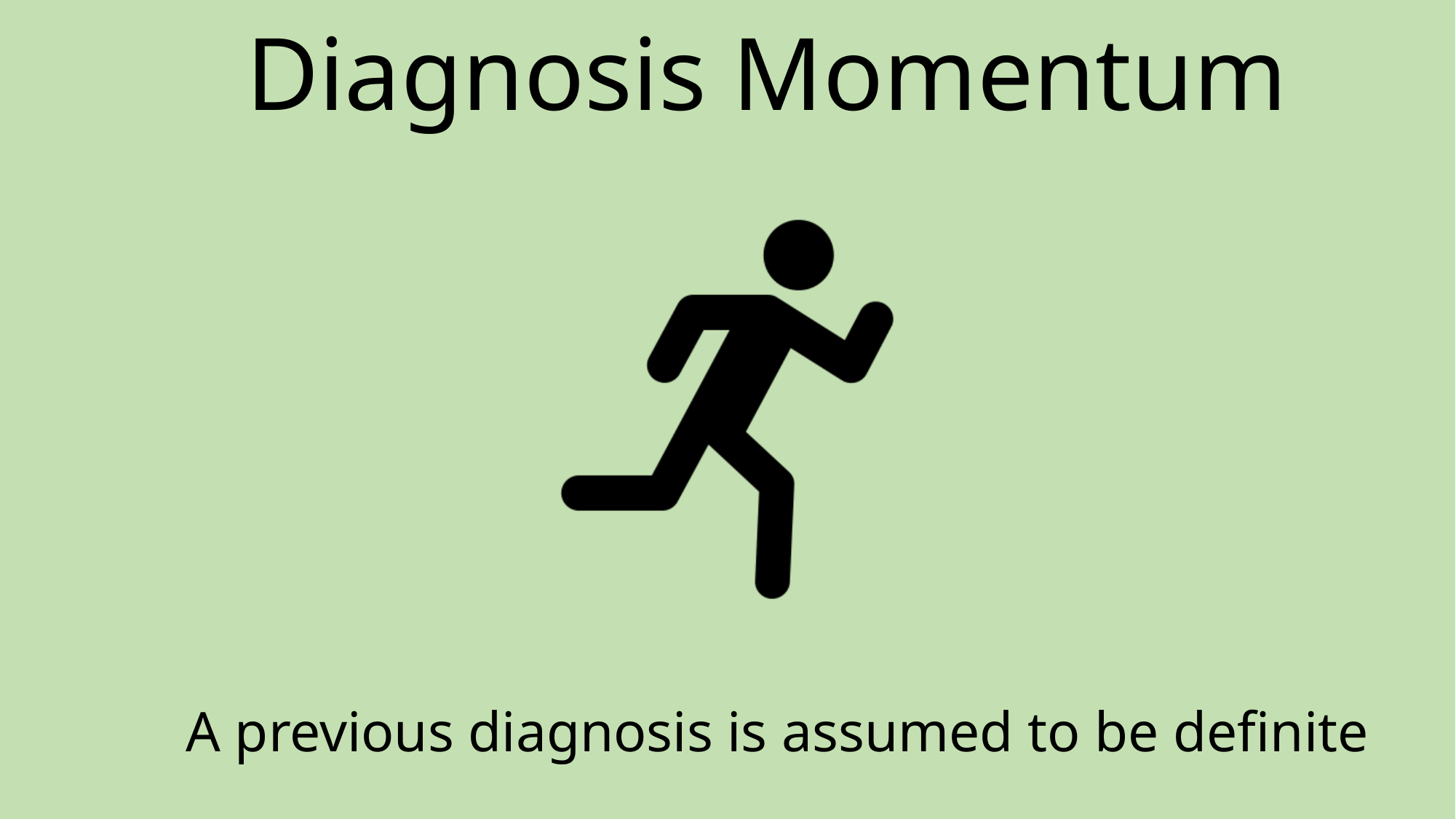

# Diagnosis Momentum
A previous diagnosis is assumed to be definite

## Slide 19
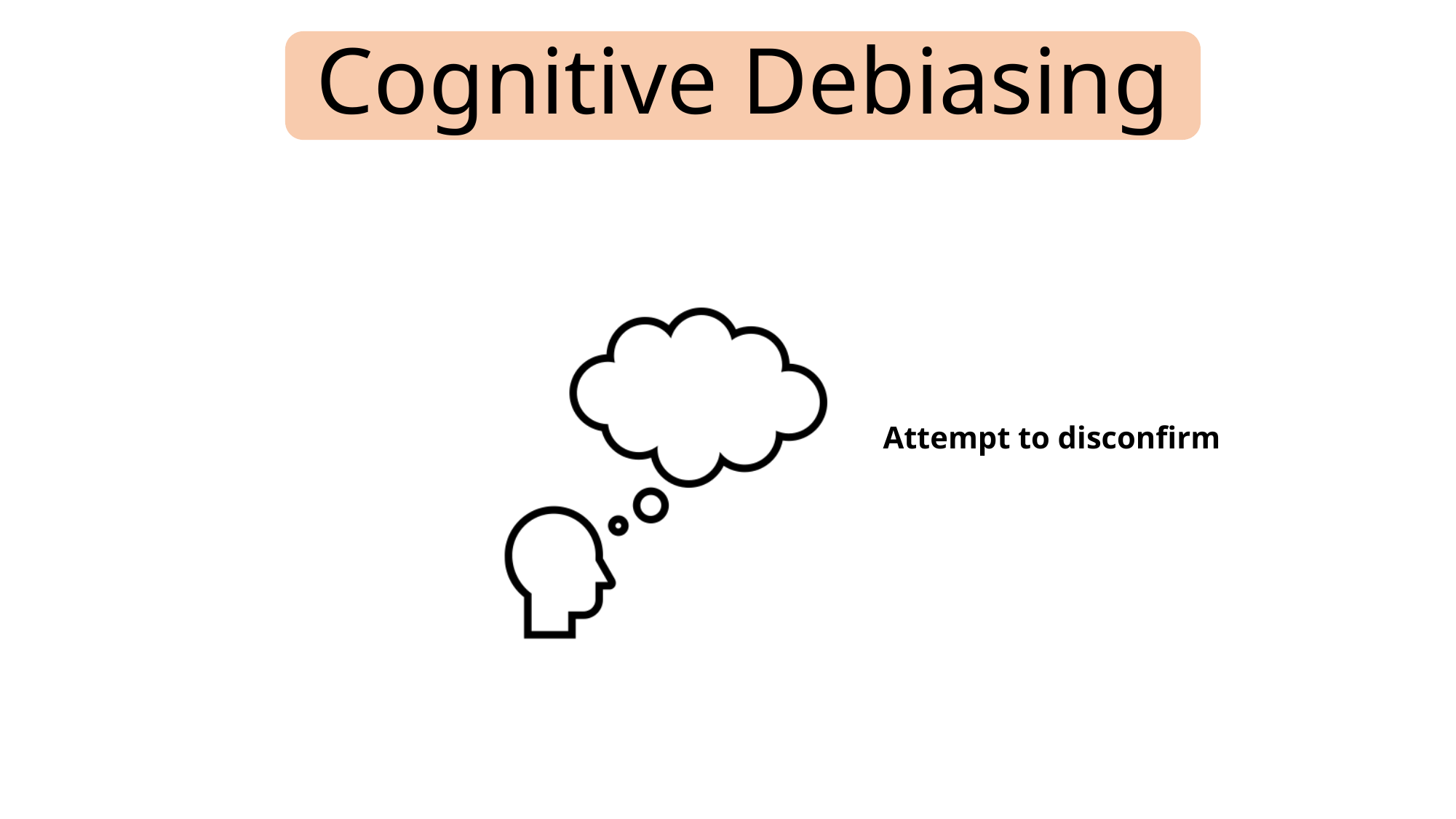

# Cognitive Debiasing
Attempt to disconfirm

## Slide 20
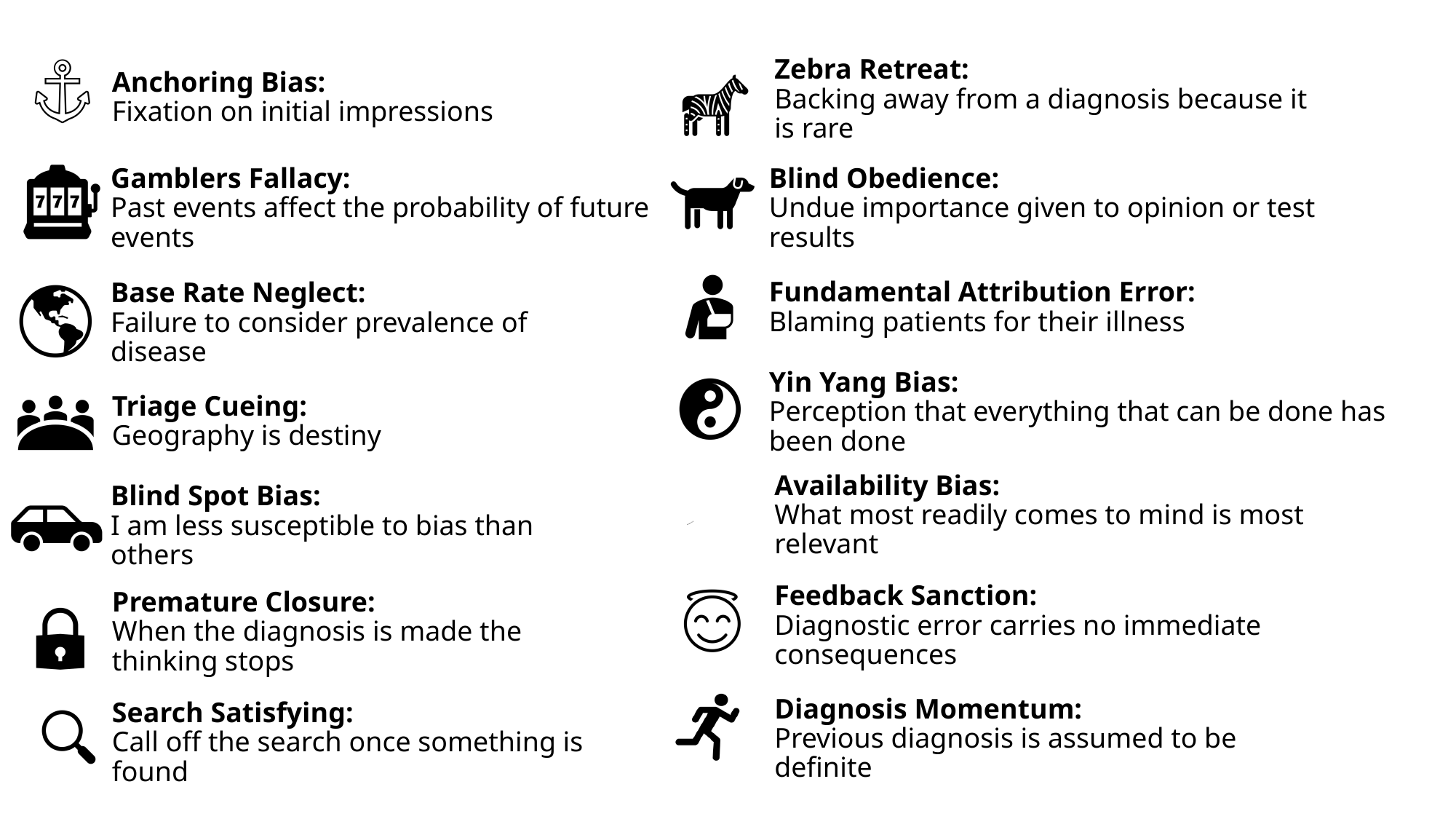

# Anchoring Bias: Fixation on initial impressions
Zebra Retreat:
Backing away from a diagnosis because it is rare
Gamblers Fallacy:
Past events affect the probability of future events
Blind Obedience:
Undue importance given to opinion or test results
Fundamental Attribution Error:
Blaming patients for their illness
Base Rate Neglect:
Failure to consider prevalence of disease
Yin Yang Bias:
Perception that everything that can be done has been done
Triage Cueing:
Geography is destiny
Availability Bias:
What most readily comes to mind is most relevant
Blind Spot Bias:
I am less susceptible to bias than others
AVAILABLE NOW!
Feedback Sanction:
Diagnostic error carries no immediate consequences
Premature Closure:
When the diagnosis is made the thinking stops
Diagnosis Momentum:
Previous diagnosis is assumed to be definite
Search Satisfying:
Call off the search once something is found

## Slide 21
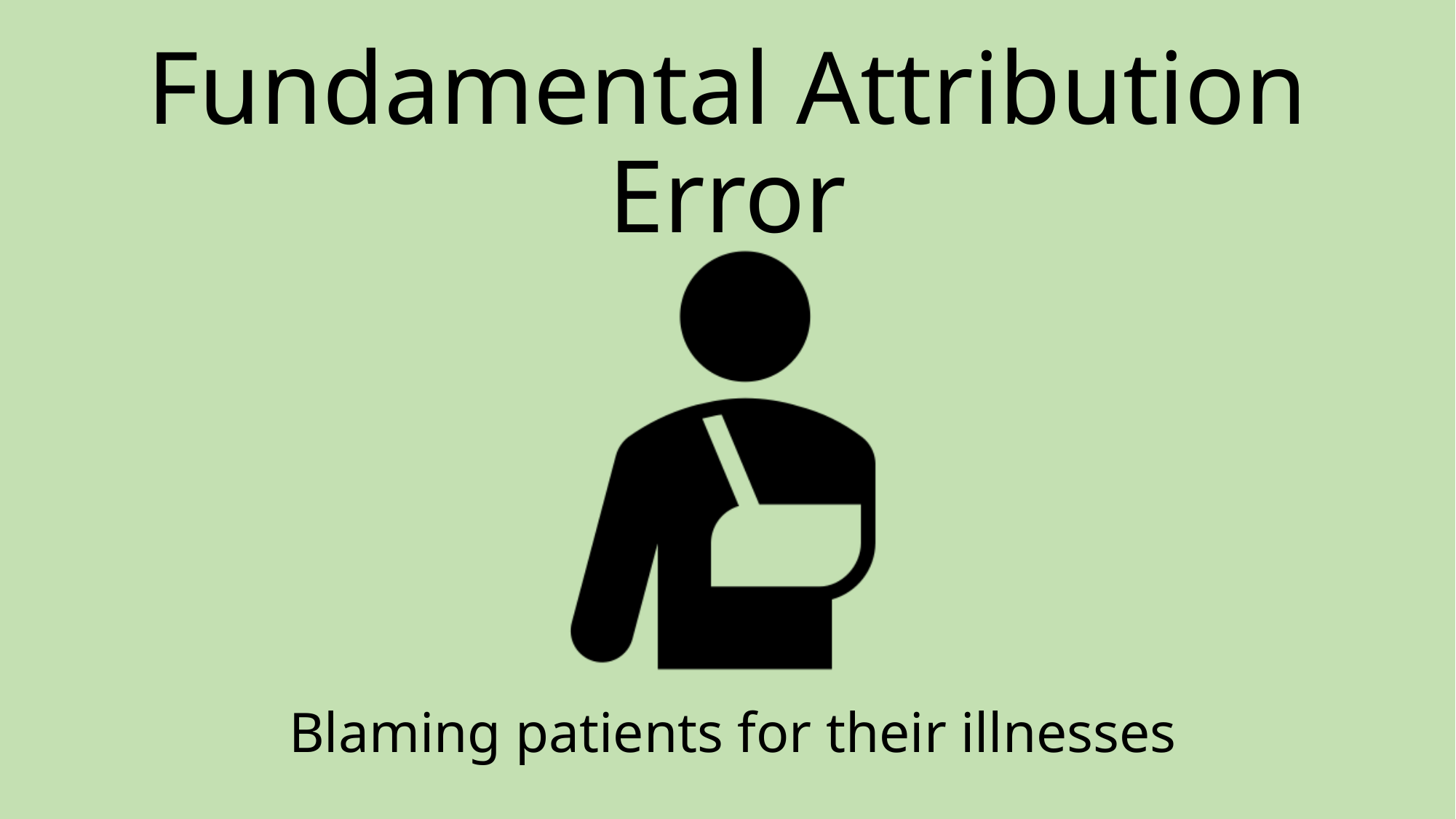

# Fundamental Attribution Error
Blaming patients for their illnesses

## Slide 22
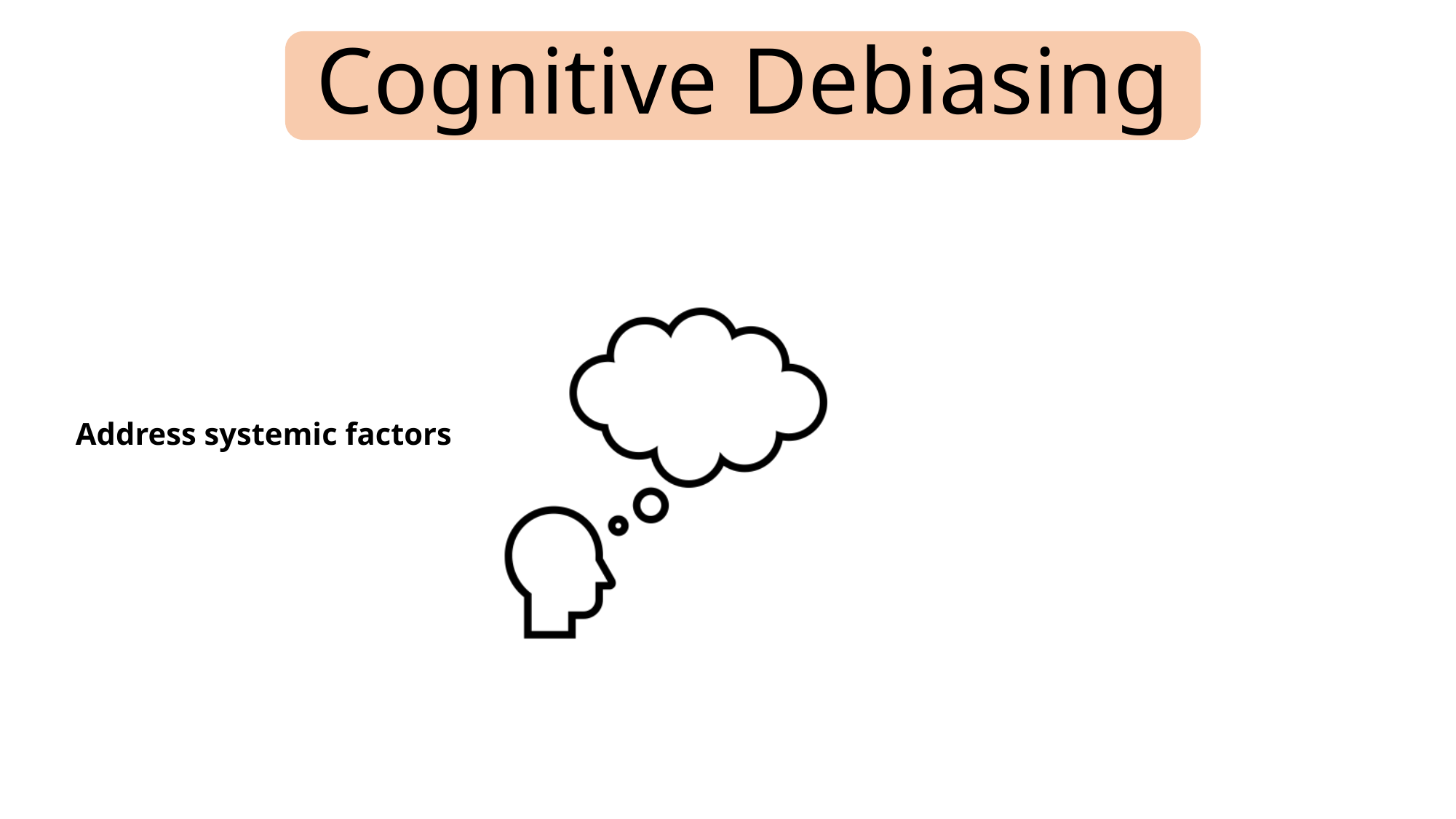

# Cognitive Debiasing
Address systemic factors

## Slide 23
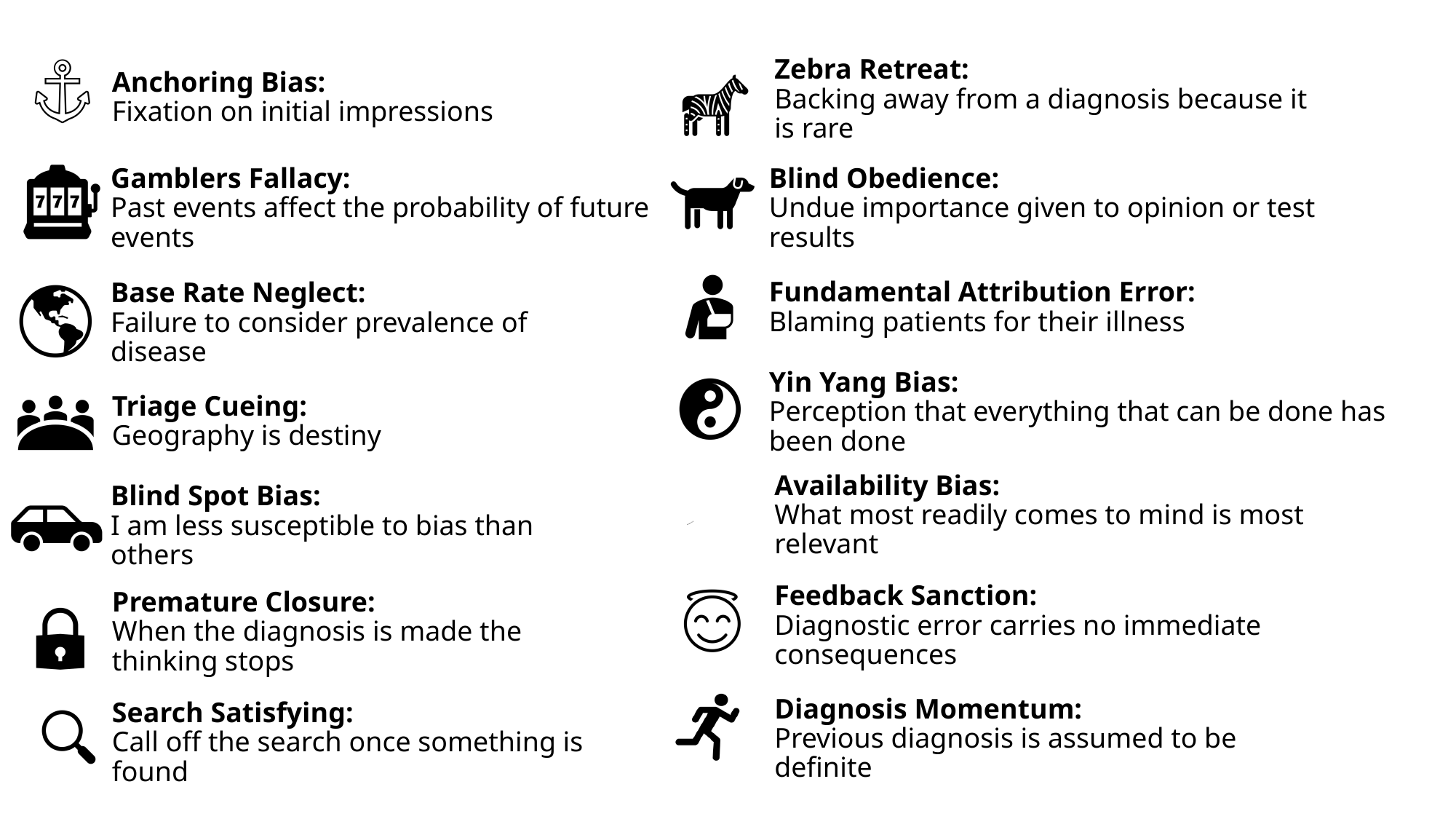

# Anchoring Bias: Fixation on initial impressions
Zebra Retreat:
Backing away from a diagnosis because it is rare
Gamblers Fallacy:
Past events affect the probability of future events
Blind Obedience:
Undue importance given to opinion or test results
Fundamental Attribution Error:
Blaming patients for their illness
Base Rate Neglect:
Failure to consider prevalence of disease
Yin Yang Bias:
Perception that everything that can be done has been done
Triage Cueing:
Geography is destiny
Availability Bias:
What most readily comes to mind is most relevant
Blind Spot Bias:
I am less susceptible to bias than others
AVAILABLE NOW!
Feedback Sanction:
Diagnostic error carries no immediate consequences
Premature Closure:
When the diagnosis is made the thinking stops
Diagnosis Momentum:
Previous diagnosis is assumed to be definite
Search Satisfying:
Call off the search once something is found

## Slide 24
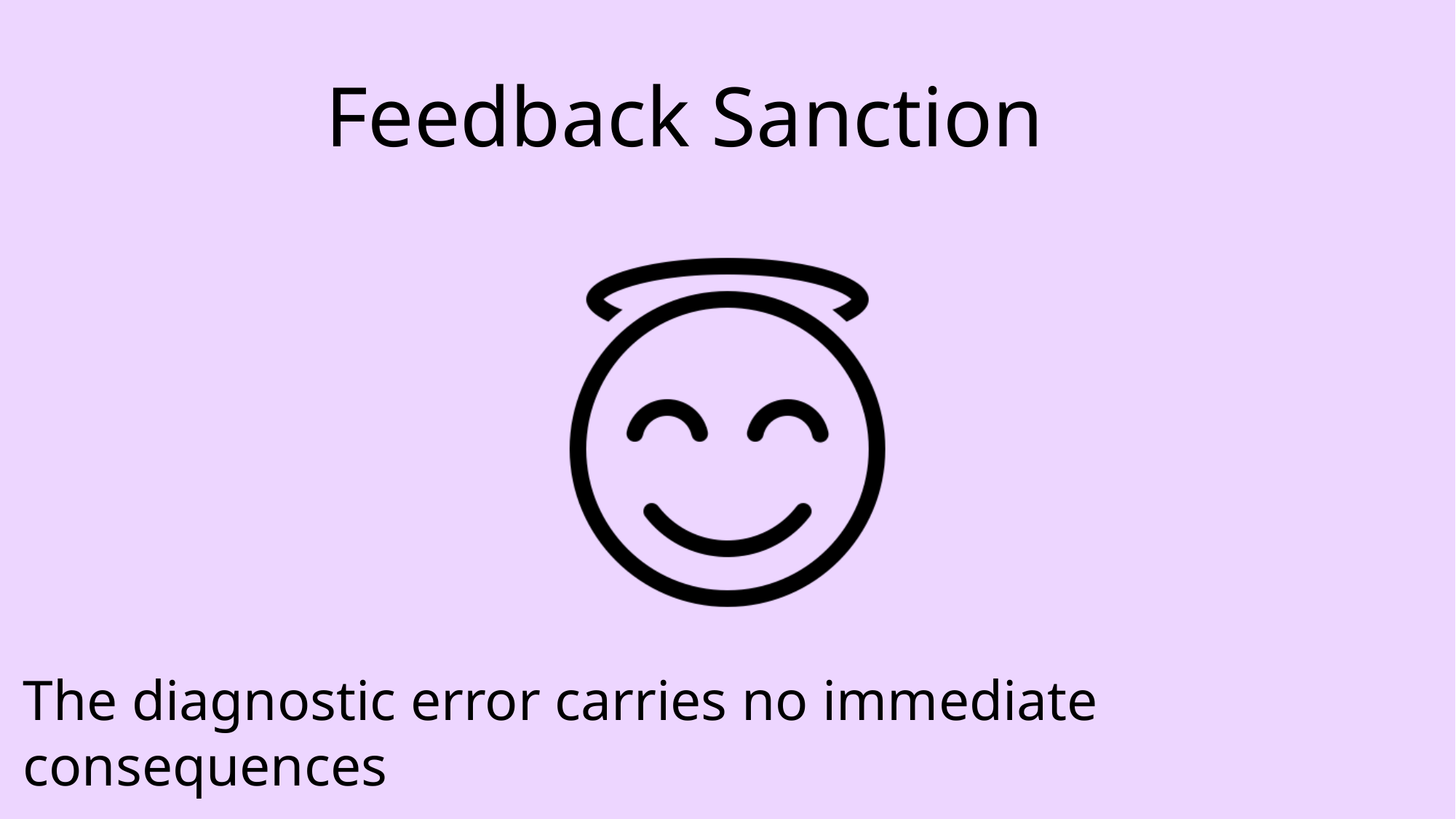

# Feedback Sanction
The diagnostic error carries no immediate consequences

## Slide 25
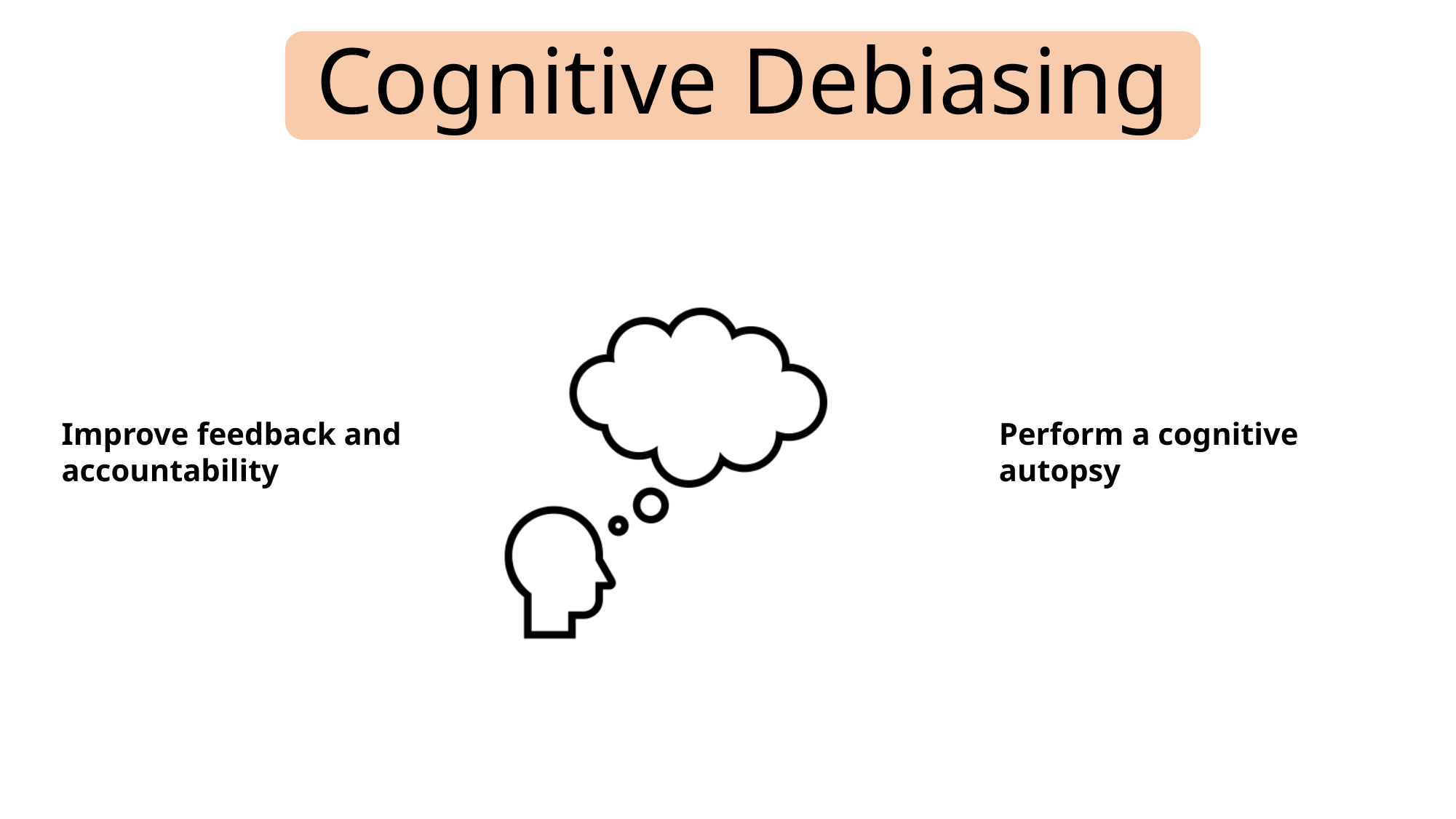

# Cognitive Debiasing
Improve feedback and accountability
Perform a cognitive autopsy

## Slide 26
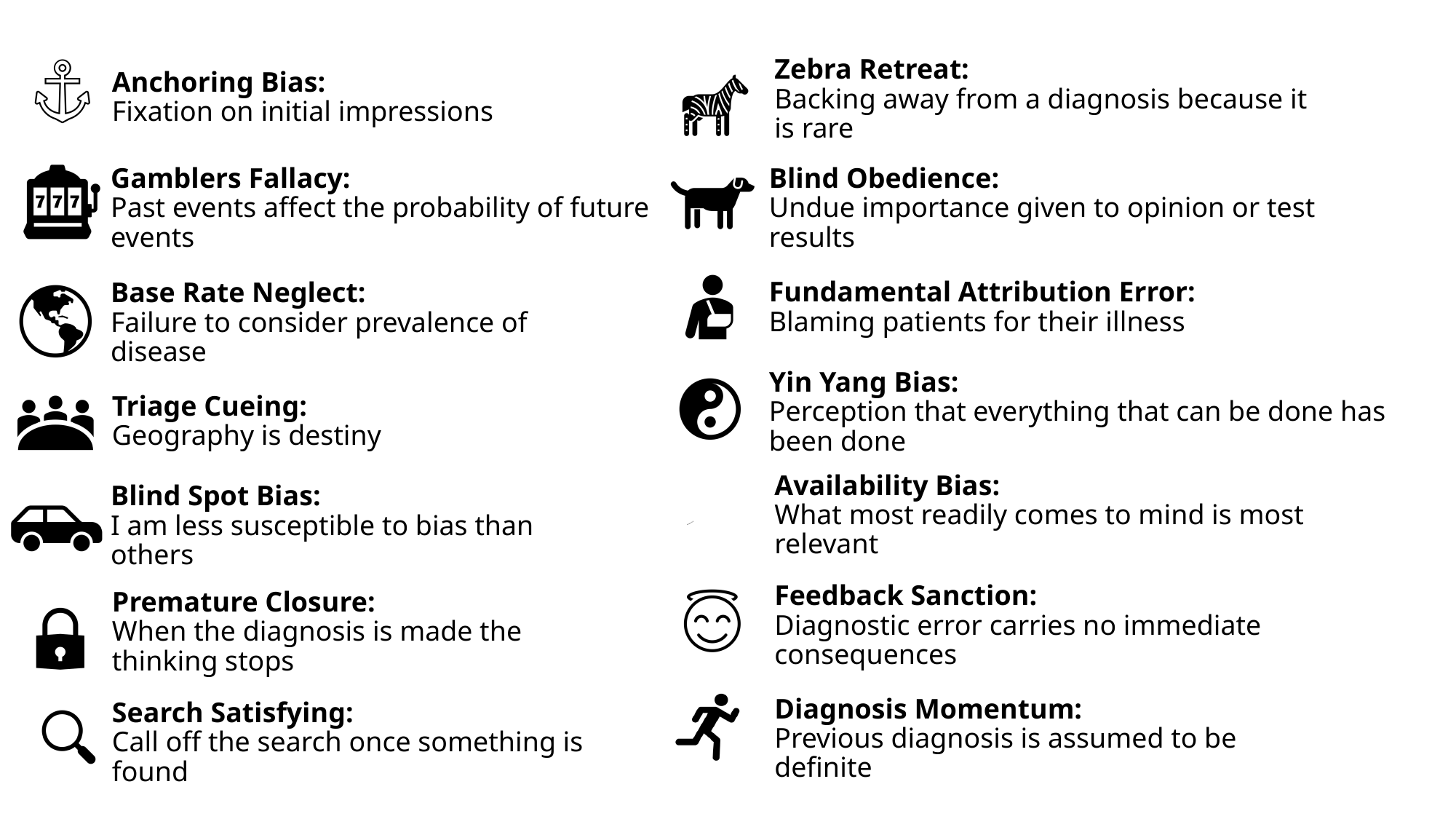

# Anchoring Bias: Fixation on initial impressions
Zebra Retreat:
Backing away from a diagnosis because it is rare
Gamblers Fallacy:
Past events affect the probability of future events
Blind Obedience:
Undue importance given to opinion or test results
Fundamental Attribution Error:
Blaming patients for their illness
Base Rate Neglect:
Failure to consider prevalence of disease
Yin Yang Bias:
Perception that everything that can be done has been done
Triage Cueing:
Geography is destiny
Availability Bias:
What most readily comes to mind is most relevant
Blind Spot Bias:
I am less susceptible to bias than others
AVAILABLE NOW!
Feedback Sanction:
Diagnostic error carries no immediate consequences
Premature Closure:
When the diagnosis is made the thinking stops
Diagnosis Momentum:
Previous diagnosis is assumed to be definite
Search Satisfying:
Call off the search once something is found

## Slide 27
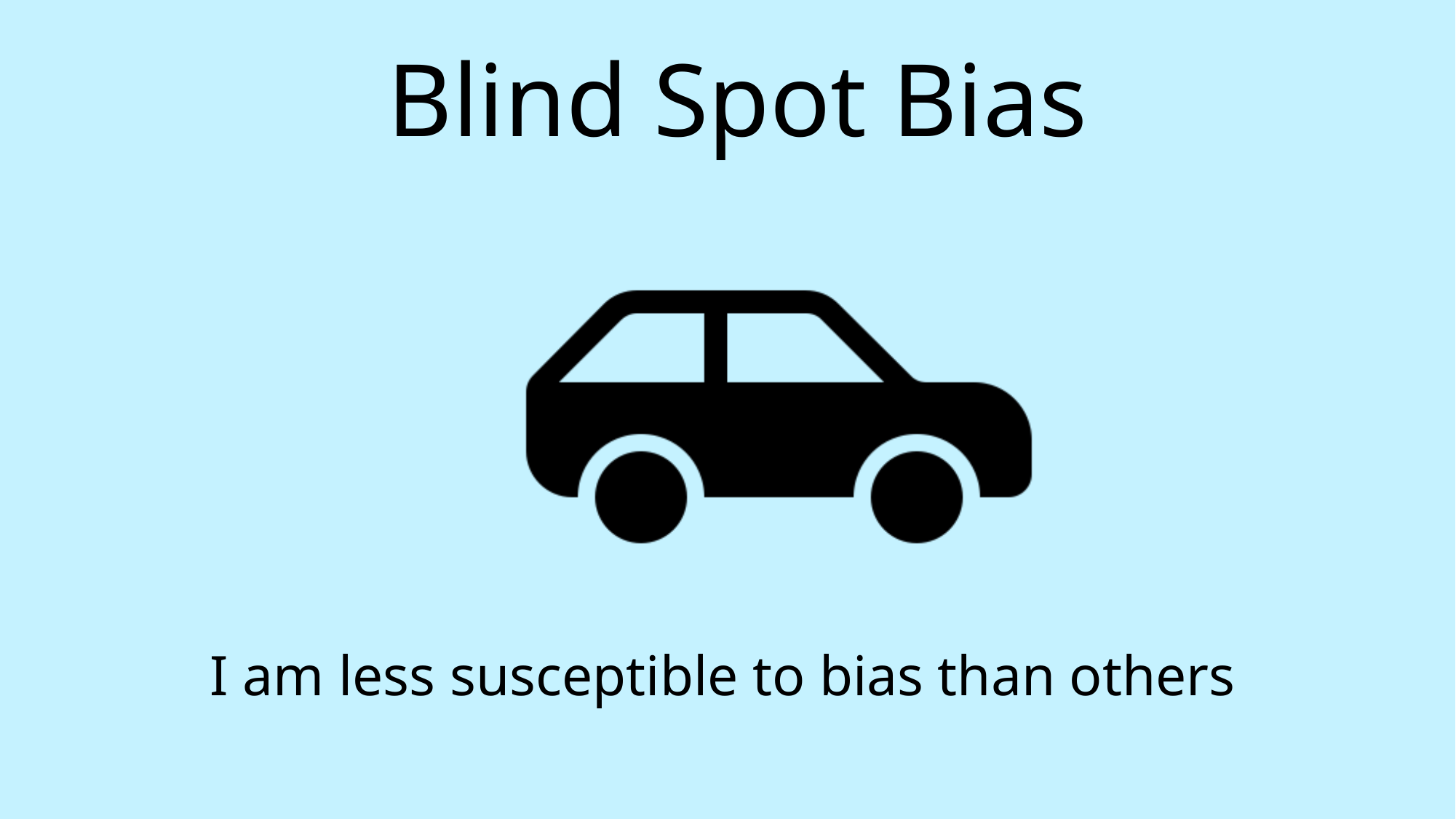

# Blind Spot Bias
I am less susceptible to bias than others

## Slide 28
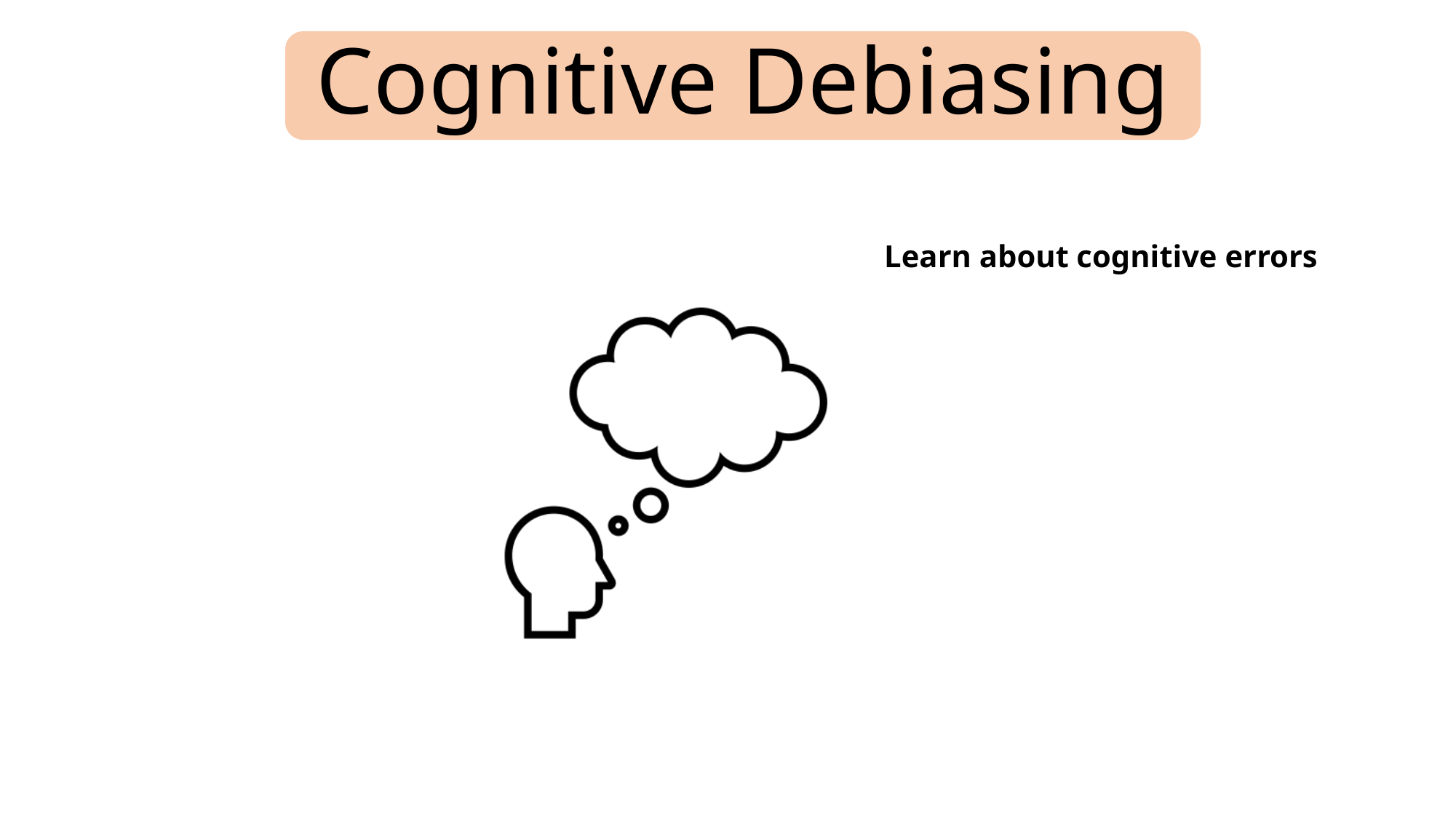

# Cognitive Debiasing
Learn about cognitive errors

## Slide 29
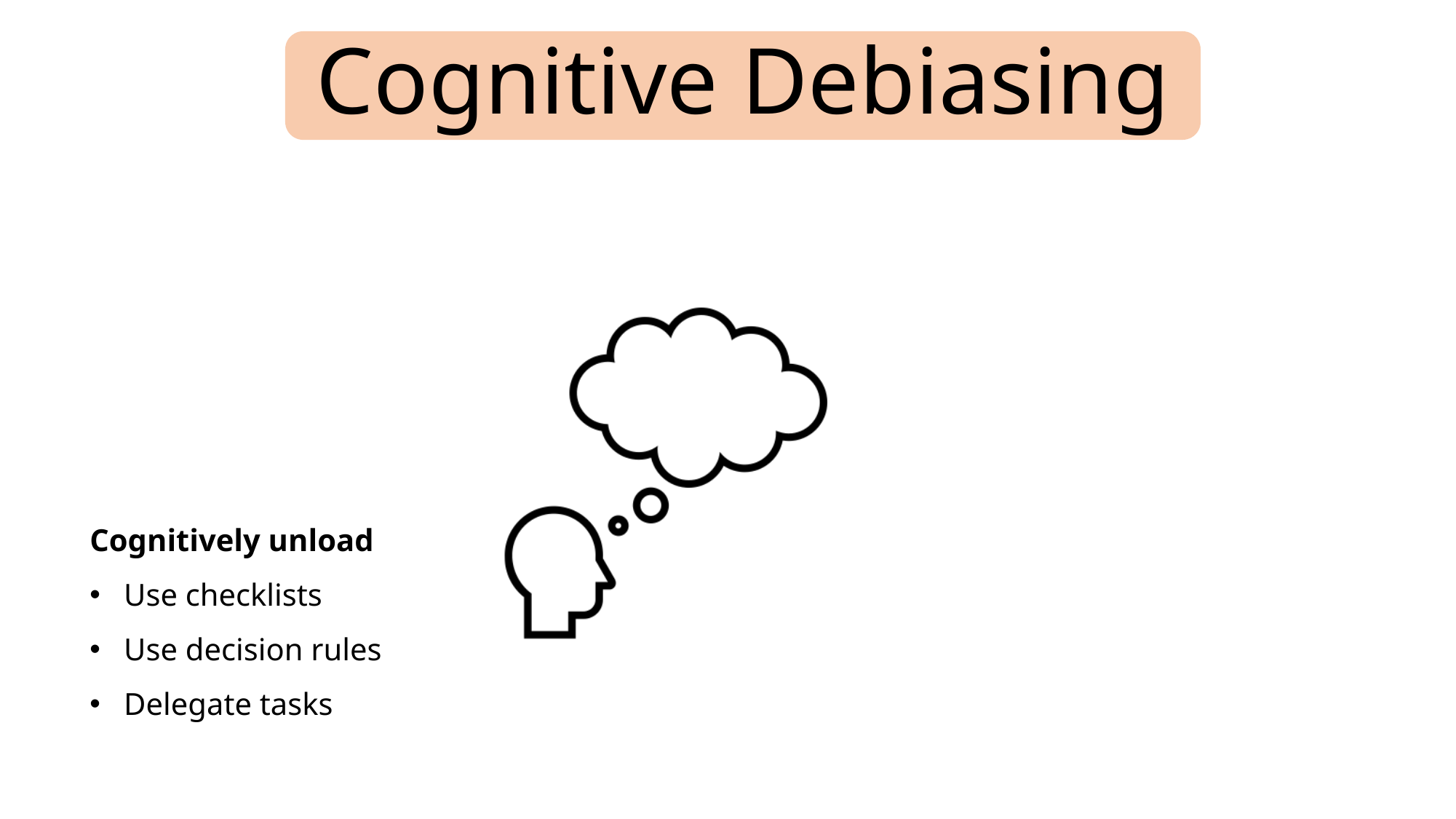

# Cognitive Debiasing
Cognitively unload
Use checklists
Use decision rules
Delegate tasks

## Slide 30
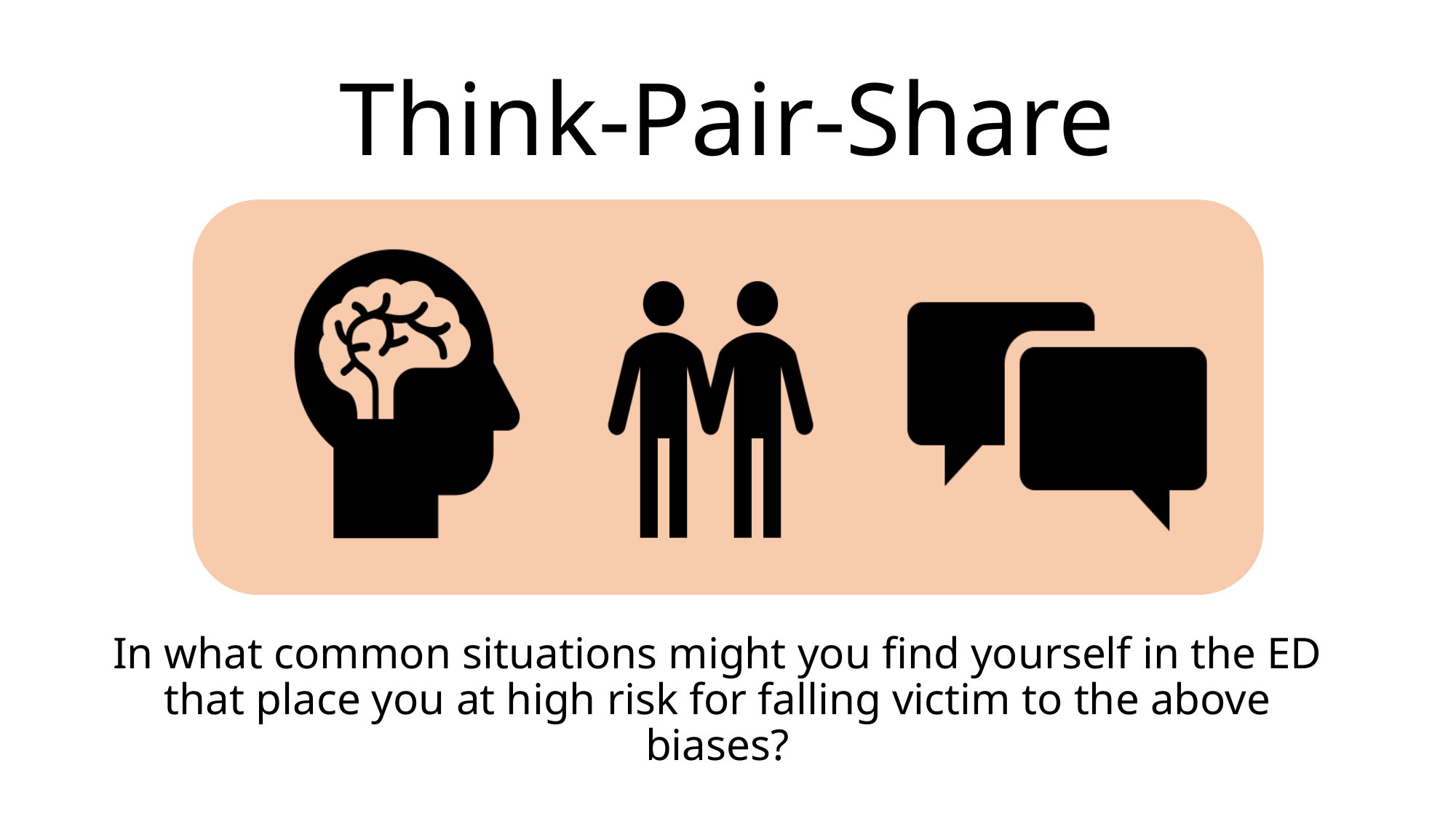

# Think-Pair-Share
In what common situations might you find yourself in the ED that place you at high risk for falling victim to the above biases?

## Slide 31
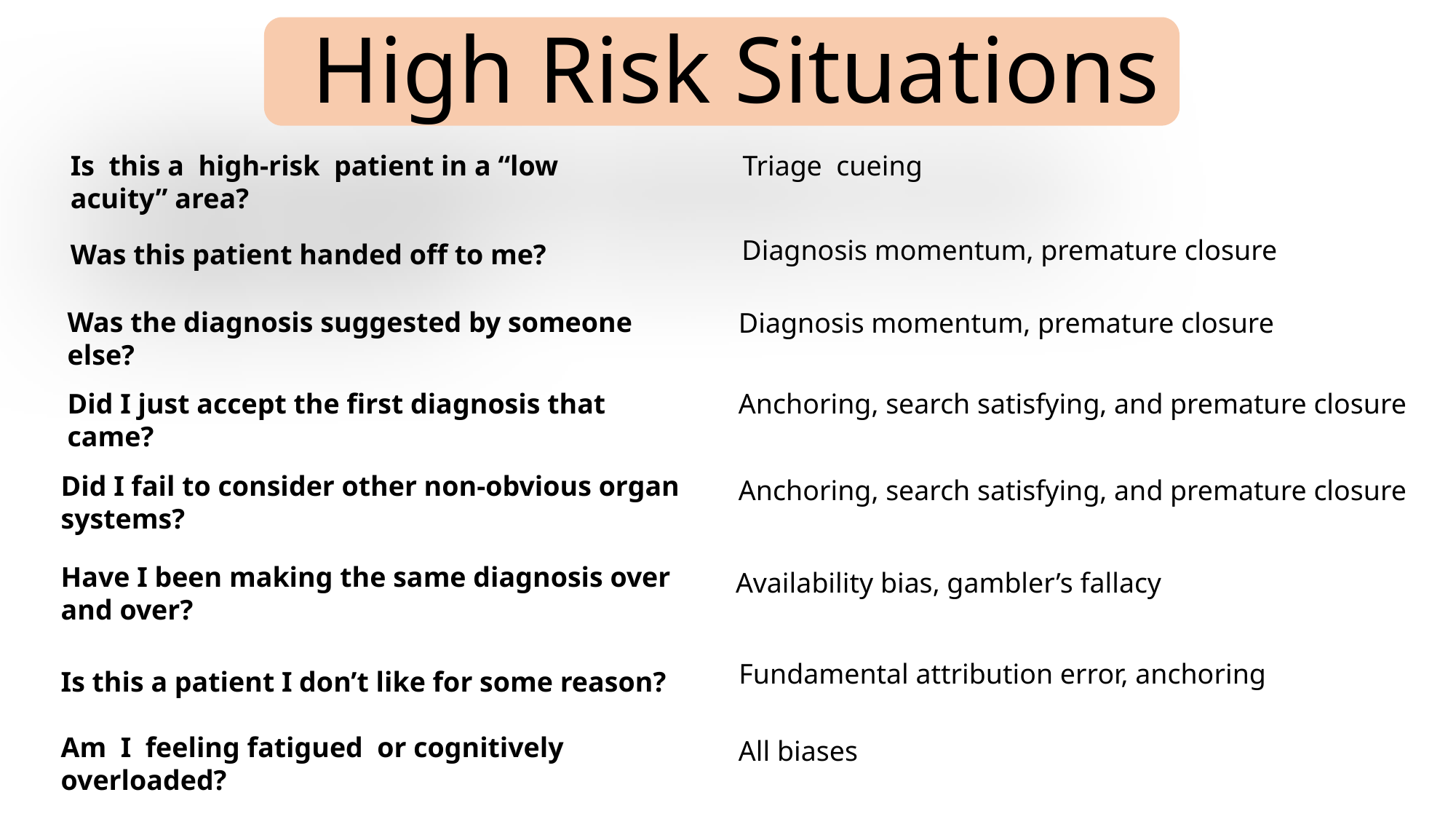

High Risk Situations
Is this a high-risk patient in a “low acuity” area?
 Triage cueing
Diagnosis momentum, premature closure
Was this patient handed off to me?
Was the diagnosis suggested by someone else?
Diagnosis momentum, premature closure
Anchoring, search satisfying, and premature closure
Did I just accept the first diagnosis that came?
Did I fail to consider other non-obvious organ systems?
Anchoring, search satisfying, and premature closure
Have I been making the same diagnosis over and over?
Availability bias, gambler’s fallacy
Fundamental attribution error, anchoring
Is this a patient I don’t like for some reason?
Am I feeling fatigued or cognitively overloaded?
All biases

## Slide 32
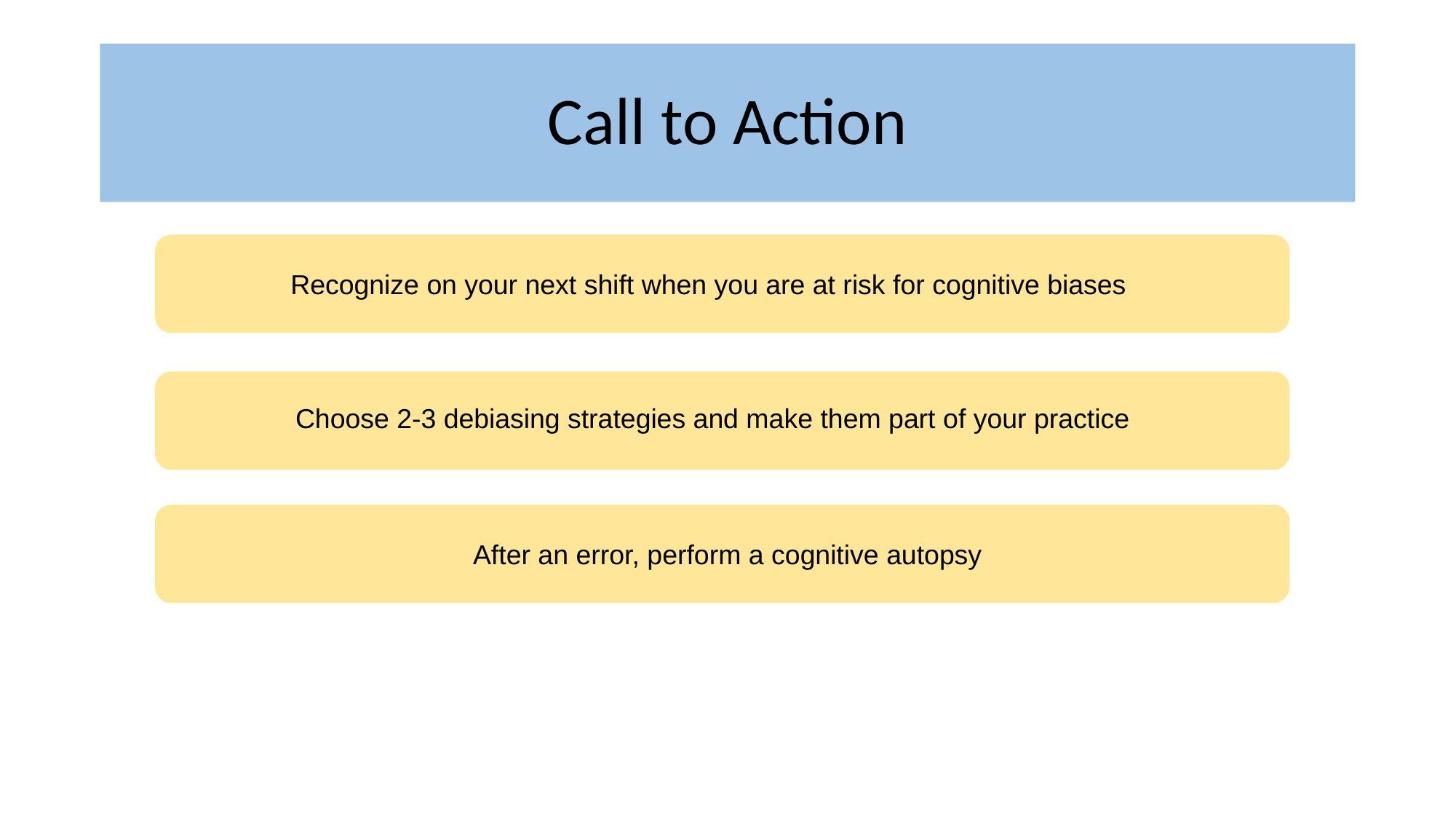

# Call to Action
Recognize on your next shift when you are at risk for cognitive biases
Choose 2-3 debiasing strategies and make them part of your practice
After an error, perform a cognitive autopsy
